# Molecular phylogeny of the family Rhabdiasidae (Nematoda: Rhabditida), with morphology, genetic characterization and mitochondrial genomes of *Rhabdias kafunata* and *R. bufonis*

**DOI:** 10.1186/s13071-024-06201-z

**Published:** 2024-03-01

**Authors:** Jia-Lu Zeng, Hui-Xia Chen, Xue-Feng Ni, Jia-Yi Kang, Liang Li

**Affiliations:** 1 Hebei Key Laboratory of Animal Physiology, Biochemistry and Molecular Biology; Hebei Collaborative Innovation Center for Eco‐Environment; College of Life Sciences, Hebei Normal University, Shijiazhuang, 050024 Hebei People’s Republic of China; 2 Hebei Research Center of the Basic Discipline Cell Biology; Ministry of Education Key Laboratory of Molecular and Cellular Biology, Shijiazhuang, 050024 Hebei People’s Republic of China

**Keywords:** Nematoda, Rhabdiasidae, Morphology, Integrative taxonomy, Mitochondrial genome, Phylogeny, Amphibian

## Abstract

**Background:**

The family Rhabdiasidae (Nematoda: Rhabditida) is a globally distributed group of nematode parasites, with over 110 species parasitic mainly in amphibians and reptiles. However, the systematic position of the family Rhabdiasidae in the order Rhabditida remains unsolved, and the evolutionary relationships among its genera are still unclear. Moreover, the present knowledge of the mitochondrial genomes of rhabdiasids remains limited.

**Methods:**

Two rhabdiasid species: *Rhabdias kafunata* Sata, Takeuchi & Nakano, 2020 and *R. bufonis* (Schrank, 1788) collected from the Asiatic toad *Bufo gargarizans* Cantor (Amphibia: Anura) in China, were identified based on morphology (light and scanning electron microscopy) and molecular characterization (sequencing of the nuclear 28S and ITS regions and mitochondrial *cox1* and *12S* genes). The complete mitochondrial genomes of *R. kafunata* and *R. bufonis* were also sequenced and annotated for the first time. Moreover, phylogenetic analyses based on the amino acid sequences of 12 protein-coding genes (PCGs) of the mitochondrial genomes were performed to clarify the systematic position of the family Rhabdiasidae in the order Rhabditida using maximum likelihood (ML) and Bayesian inference (BI). The phylogenetic analyses based on the 28S + ITS sequences, were also inferred to assess the evolutionary relationships among the genera within Rhabdiasidae.

**Results:**

The detailed morphology of the cephalic structures, vulva and eggs in *R. kafunata* and *R. bufonis* was revealed using scanning electron microscopy (SEM) for the first time. The characterization of 28S and ITS regions of *R. kafunata* was reported for the first time. The mitogenomes of *R. kafunata* and *R. bufonis* are 15,437 bp and 15,128 bp long, respectively, and both contain 36 genes, including 12 PCGs (missing *atp8*). Comparative mitogenomics revealed that the gene arrangement of *R. kafunata* and *R. bufonis* is different from all of the currently available mitogenomes of nematodes. Phylogenetic analyses based on the ITS + 28S data showed *Neoentomelas* and *Kurilonema* as sister lineages, and supported the monophyly of *Entomelas*, *Pneumonema*, *Serpentirhabdias* and *Rhabdias*. Mitochondrial phylogenomic results supported Rhabdiasidae as a member of the superfamily Rhabditoidea in the suborder Rhabditina, and its occurrance as sister to the family Rhabditidae.

**Conclusions:**

The complete mitochondrial genome of *R. kafunata* and *R. bufonis* were reported for the first time, and two new gene arrangements of mitogenomes in Nematoda were revealed. Mitogenomic phylogenetic results indicated that the family Rhabdiasidae is a member of Rhabditoidea in Rhabditina, and is closely related to Rhabditidae. Molecular phylogenies based on the ITS + 28S sequence data supported the validity of *Kurilonema*, and showed that *Kurilonema* is sister to *Neoentomelas*. The present phylogenetic results also indicated that the ancestors of rhabdiasids seem to have initially infected reptiles, then spreading to amphibians.

**Graphical Abstract:**

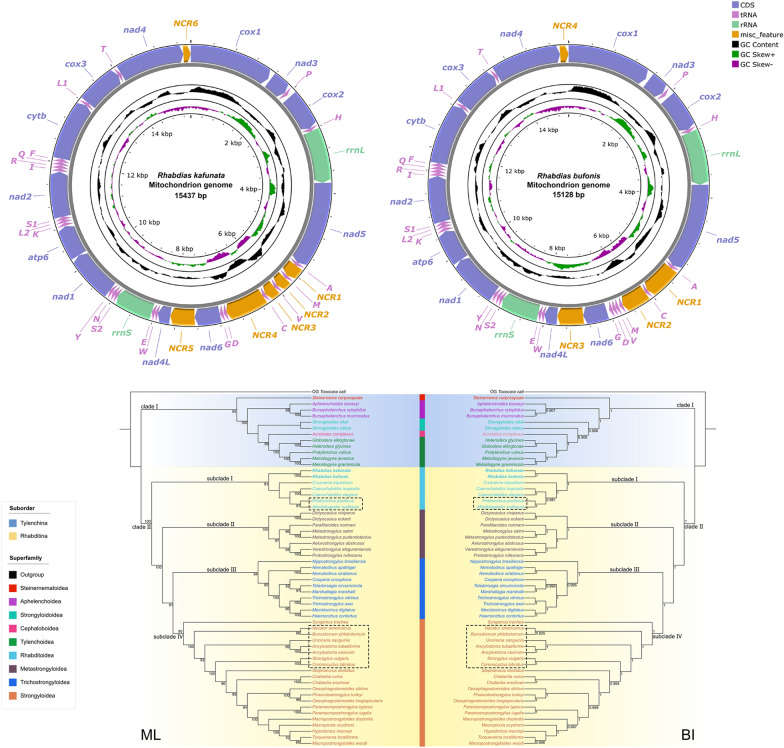

**Supplementary Information:**

The online version contains supplementary material available at 10.1186/s13071-024-06201-z.

## Background

The family Rhabdiasidae (Nematoda: Rhabditida) currently includes eight genera, namely *Rhabdias*, *Entomelas*, *Kurilonema*, *Neoentomelas*, *Chabirenia*, *Acanthorhabdias*, *Pneumonema* and *Serpentirhabdias*, with over 110 species mostly parasitic in amphibians and reptiles worldwide [1–5]. However, the evolutionary relationships of these eight genera remain unclear. In recent years, large numbers of the complete mitochondrial genomes (mitogenomes) of nematodes have been sequenced and used for phylogeny, population genetics and evolutionary history investigations [6–8]. However, the present knowledge of the mitochondrial genomes of rhabdiasids remains limited.

Although some studies made important attempts to reconstruct the molecular phylogeny of Nematoda [9–20], the systematic position of the family Rhabdiasidae in the order Rhabditida is still unclear. Anderson & Bain [21] put Rhabdiasidae, closer to the families Strongyloididae, Rhabditidae, Cylindrocorporidae and Cephalobidae within the superfamily Rhabditoidea (order Rhabditida). However, De Ley & Blaxter [10, 11] placed the Rhabdiasidae, Strongyloididae and Steinernematidae in the superfamily Strongyloidoidea (suborder Tylenchina) [10, 11]. Hodda [22, 23] erected the superfamily Steinernematoidea to allocate Steinernematidae, and transferred Strongyloidoidea (which includes Rhabdiasidae and Strongyloididae) and Steinernematoidea to Panagrolaimina [22, 23].

In the present study, to enrich the mitogenomic data and investigate the patterns of mitogenomic evolution of the family Rhabdiasidae, the complete mitochondrial genomes of *Rhabdias kafunata* Sata, Takeuchi & Nakano, 2020 and *R. bufonis* (Schrank, 1788) were sequenced and annotated for the first time. Moreover, to assess the evolutionary relationships among the genera within Rhabdiasidae and clarify the systematic position of the Rhabdiasidae in Rhabditida, phylogenetic analyses based on the nuclear large ribosomal subunit (28S) and internal transcribed spacer (ITS) sequences, and the amino acid sequences of 12 protein-coding genes (PCGs) of mitochondrial genomes, were conducted using maximum likelihood (ML) and Bayesian inference (BI).

## Methods

### Parasite collection and species identification

Large numbers of nematodes belonging to the genus *Rhabdias* were collected from the lung of the Asiatic toad *Bufo gargarizans* Cantor (Amphibia: Anura) in Shijiazhuang, Hebei Province, China, fixed and stored in 80% ethanol until study. These nematodes were identified to the species level using light and scanning electron microscopy, in addition to the genetic characterization of the nuclear 28S and ITS and mitochondrial cytochrome *c* oxidase subunit 1 (*cox*1) and 12S small subunit ribosomal RNA gene.

For light microscopy, nematodes were cleared in glycerin, and then examined and photographed using a Nikon® optical microscope (Nikon ECLIPSE Ni-U, Nikon corporation, Tokyo, Japan). For scanning electron microscopy (SEM), the anterior and posterior ends of nematodes were transferred to 4% formaldehyde solution, and then post-fixed in 1% O_s_O_4_, dehydrated via an ethanol series and acetone and critical point dried. The specimens were coated with gold and examined using a Hitachi S-4800 scanning electron microscope (Hitachi Ltd., Tokyo, Japan) at an accelerating voltage of 20 kV. Voucher specimens were deposited in College of Life Sciences, Hebei Normal University, Hebei Province, China.

For molecular procedures, a total of nine nematode specimens (four individuals of *R. kafunata* and five individuals of *R. bufonis*) were randomly selected for the following procedures. Genomic DNA from each individual was extracted using a Column Genomic DNA Isolation Kit (Shanghai Sangon, China) according to the manufacturer’s instructions. DNA was eluted in elution buffer and kept it at −20 °C until use. The primers and cycling conditions for amplifying different target regions by polymerase chain reaction (PCR) are provided in Additional file 1: Table S1. All PCR reactions were performed in 50 μl consisting of 10 mM Tris HCl at pH 8.4, 50 mM KCl, 3.0 mM MgCl_2_, 250 μM of each dNTP, 50 pmol of each primer, and 1.5 U of Taq polymerase (Takara Bio Inc., Kusatsu, Shiga, Japan) in a thermocycler (model 2720; Applied Biosystems, Thermo Fisher Scientific, Waltham, MA, USA).

PCR products were checked on GoldView-stained 1.5% agarose gel and purified by the Column PCR Product Purification Kit (Shanghai Sangon, China). Sequencing for each sample was carried out for both strands using a DyeDeoxyTerminator Cycle Sequencing Kit (v.2, Applied Biosystems, California, USA). Sequences of *R. kafunata* and *R. bufonis* obtained herein were aligned and compared with genetic data of *Rhabdias* available in the National Center for Biotechnology Information (NCBI) database (http://www.ncbi.nlm.nih.gov) using ClustalW2. The 28S, ITS, *cox*1 and 12S sequences of *R. kafunata* and *R. bufonis* were deposited in the GenBank database (http://www.ncbi.nlm.nih.gov).

### Mitochondrial genome sequencing, assembly, and annotation

A total of 30 Gb clean genomic data of each species were generated using the Pair-End 150 sequencing method on the Illumina NovaSeq 6000 platform by Novogene (Tianjin, China). The complete mitochondrial genomes were assembled using GetOrganelle v1.7.2a [24]. Protein coding genes (PCGs), ribosomal RNA (rRNAs), and transfer RNA (tRNAs) were annotated using MitoS web server (http://mitos2.bioinf.uni-leipzig.de/index.py) and MitoZ v2.4 [25]. The open reading frame (ORF) of each PCG was confirmed manually by the web version of ORF finder (https://www.ncbi.nlm.nih.gov/orffinder/). The “lost” tRNA genes ignored by both MitoS and MitoZ were identified using BLAST on the basis of a database of the existing tRNA sequences of nematodes. The secondary structures of tRNAs were predicted by ViennaRNA module [26], building on MitoS2 [27] and RNAstructure v6.3 [28], followed by manual correction. MitoZ v2.4 was used to visualize and depict gene element features [25]. The base composition, amino acid usage, and relative synonymous codon usage (RSCU) were calculated by Python script, which refers to Codon Adaptation Index (CAI) [29]. The total length of the base composition included ambiguous bases. The base skew analysis was used to describe the base composition of nucleotide sequences. The complete mitochondrial genomes of *R. kafunata* and *R. bufonis* obtained were deposited in GenBank (http://www.ncbi.nlm.nih.gov).

### Phylogenetic analyses

Phylogenetic analyses of rhabdiasid nematodes were performed on the basis of the ITS + 28S sequences using the maximum likelihood (ML) with IQ-TREE [30] and Bayesian inference (BI) with MrBayes [31]. *Caenorhabditis elegans* (Rhabditida: Rhabditidae) was chose as the out-group. The in-group included 47 rhabdiasid species representing six genera belonging to Rhabdiasidae. Detailed information on species included in the phylogenetic analyses is provided in Additional file 2: Table S2. Genes were aligned separately using the MAFFT v7.313 multiple sequence alignment program under the iterative refinement method of E-INS-I [32]. In addition, partially ambiguous bases were manually inspected and removed. The aligned and pruned sequences were concatenated into a matrix by PhyloSuite v1.2.2. The TPM2u + F + I + I + R2 model was selected for ML analyses. The GTR + F + G4 models were selected for BI analyses. Reliabilities for ML inference were tested using 1000 BS replications, and BIC analysis was run for 5 × 10^6^ MCMC generations.

Phylogenetic analyses were also performed on the basis of concatenated amino acid sequences of 12 PCGs using maximum likelihood (ML) and Bayesian inference (BI). *Toxocara cati* (Ascaridida: Toxocaridae) was chosen as the out-group in this case. The in-group included 12 species of Tylenchina and 46 species of Rhabditina. Detailed information on the representatives included in this analysis is provided in Additional file 3: Table S3. Substitution models were compared and selected according to the Bayesian Information Criterion by using ModelFinder. For ML inference, the mtZOA + F + R6 model was identified as the optimal nucleotide substitution model. For BI inference, the optimal nucleotide substitution models selected for each partitioning scheme were provided in Additional file 4: Table S4.

In the ML tree, the bootstrap support (BS) values ≥ 80 were considered to constitute strong nodal support, whereas BS values ≥ 50 and < 80 were considered to constitute moderate nodal support. In the BI tree, Bayesian posterior probabilities (BPP) values ≥ 0.98 were considered to constitute strong nodal support, whereas BPP values ≥ 0.95 and < 0.98 were considered to constitute moderate nodal support. BS values ≥ 50 and BPP values ≥ 0.95 are shown in the phylogenetic trees.

## Results

### Species identification

#### *Rhabdias kafunata* Sata, Takeuchi & Nakano, 2020; Figs. 1–3; Table 1

**Fig. 1:**
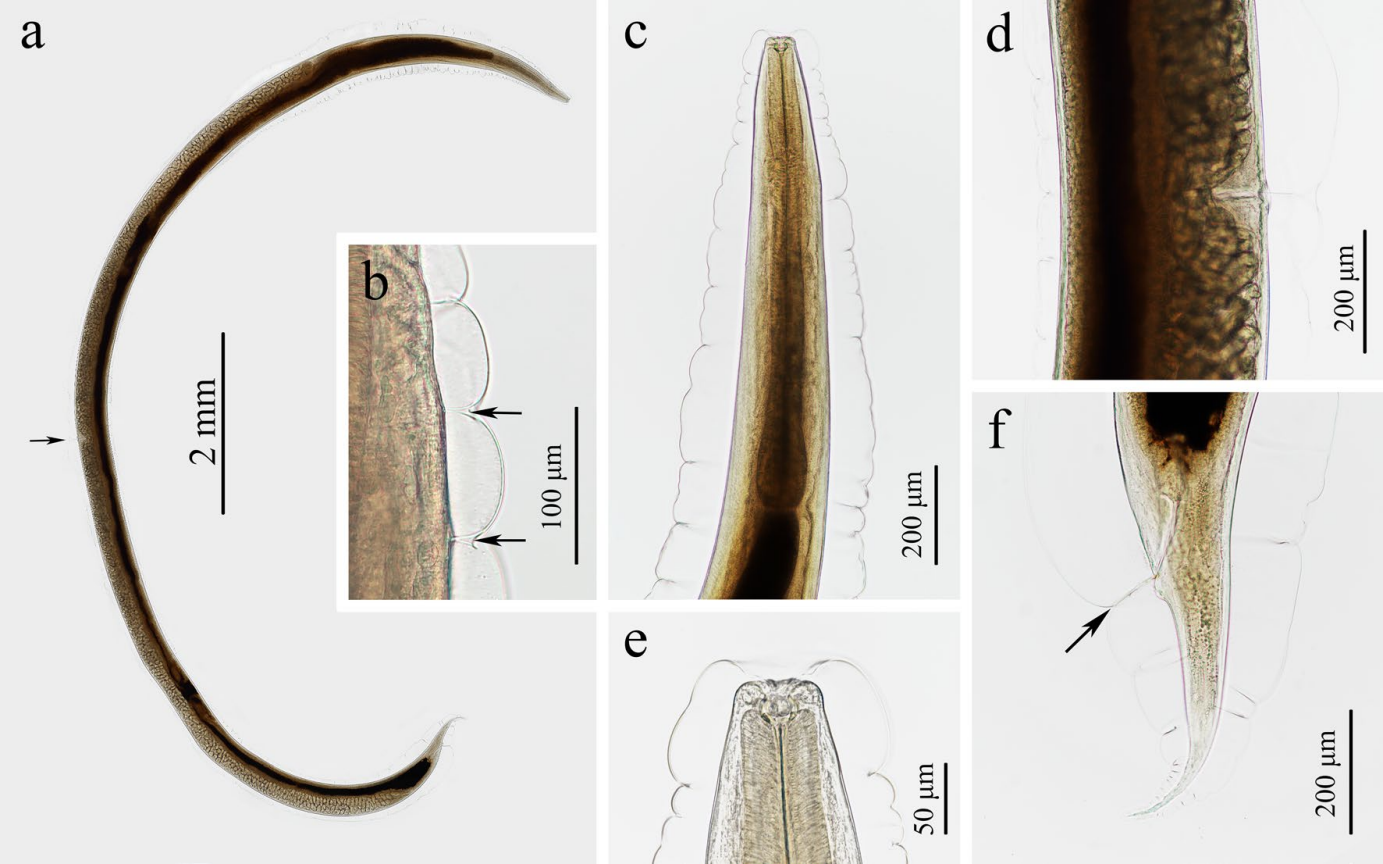
Photomicrographs of *Rhabdias kafunata* from *Bufo gargarizans* in China. **a** Entire body (arrow indicating the vulva), lateral view; **b** lateral cuticular pores (arrow), lateral view; **c** anterior part of body, lateral view; **d** region of vulva, lateral view; **e** cephalic extremity, lateral view; **f** posterior part of body (arrow indicating the cloaca), lateral view

**Table 1:** Morphometric comparisons of *Rhabdias kafunata*

Characteristics	Present study	Sata et al. (2020)
	Female	Female
BL (mm)	13.3–17.1	10.9–16.8
BMW	290–391	204–355
SBC	20.0–25.0 × 27.5–37.5	15.0–19.0 × 21.0–30.0
EL	810–900	694–818
AW	45.0–57.5	33.0–46.0
DW	52.5–67.5	47.0–59.0
GW	47.5–62.5	39.0–56.0
BW	87.5–110	80.0–101
NRC	227–266	195–259
EPC	266–386	228–277
VC (mm)	6.59–9.02	5.97–8.32
TL	314–483	376–493
SE	33.8–53.1 × 58.0–106	42.0–55.0 × 89.0–107
EL/BL (%)	5.51–7.46	4.20–7.30
VC/BL (%)	50.9–55.7	49.6–55.9
TL/BL (%)	2.24–3.47	2.80–4.30
Host	*Bufo gargarizans*	*Bufo gargarizans miyakonis*
Locality	China (Hebei Province)	Japan (Okinawa Prefecture)

Measurements are given in micrometers unless otherwise stated. BL, length of body; BMW, maximum width of body; SBC, size of buccal capsule; EL, length of entire esophagus; AW, width of anterior part of esophagus; DW, width of esophageal dilatation; GW, width of middle glandular part of esophagus; BW, width of posterior end of esophagus; NRC, distance of nerve-ring to cephalic end; EPC, distance of excretory pore to cephalic end; VC, distance of vulva to cephalic end; TL, length of tail; SE, well-developed, embryonated eggs

*Present host*: Asiatic toad *Bufo gargarizans* Cantor (Amphibia: Anura).

*Present locality*: Shijiazhuang, Hebei Province, China.

*Site in host*: Lung.

*Prevalence and intensity of infection*: 11 of 50 (22.0%) toads examined, and infected with intensity of 1–14 (mean 5.7) nematodes per infected host (a total of 63 female nematode specimens collected).

*GenBank accession:* OR682285–OR682288 (28S), OR682645–OR682648 (ITS), OR685545–OR685548 (*cox1*), OR711464–OR711466 (12S), OR725305 (mitogenome).

#### Morphological description

**Fig. 2:**
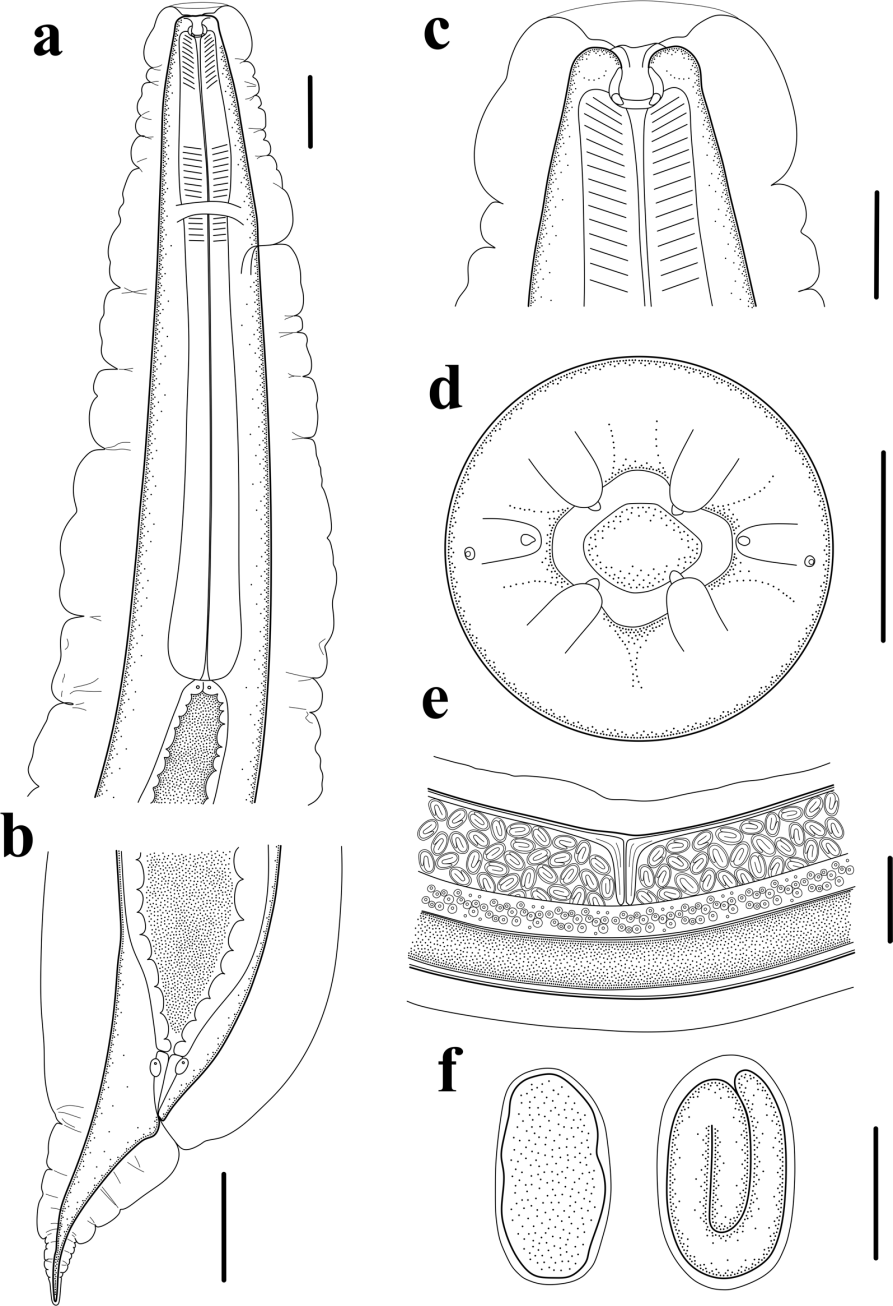
Line drawings of *Rhabdias kafunata* from *Bufo gargarizans* in China. **a** Anterior part of body, lateral view; **b** posterior part of body, lateral view; **c** cephalic extremity, lateral view; **d** cephalic extremity, apical view; **e** region of vulva, lateral view; **f** eggs. *Scale bars*: **a** 100 μm; **b**, **e**, 200 μm; **c**, **f**, 50 μm; **d**, 20 μm

**Fig. 3:**
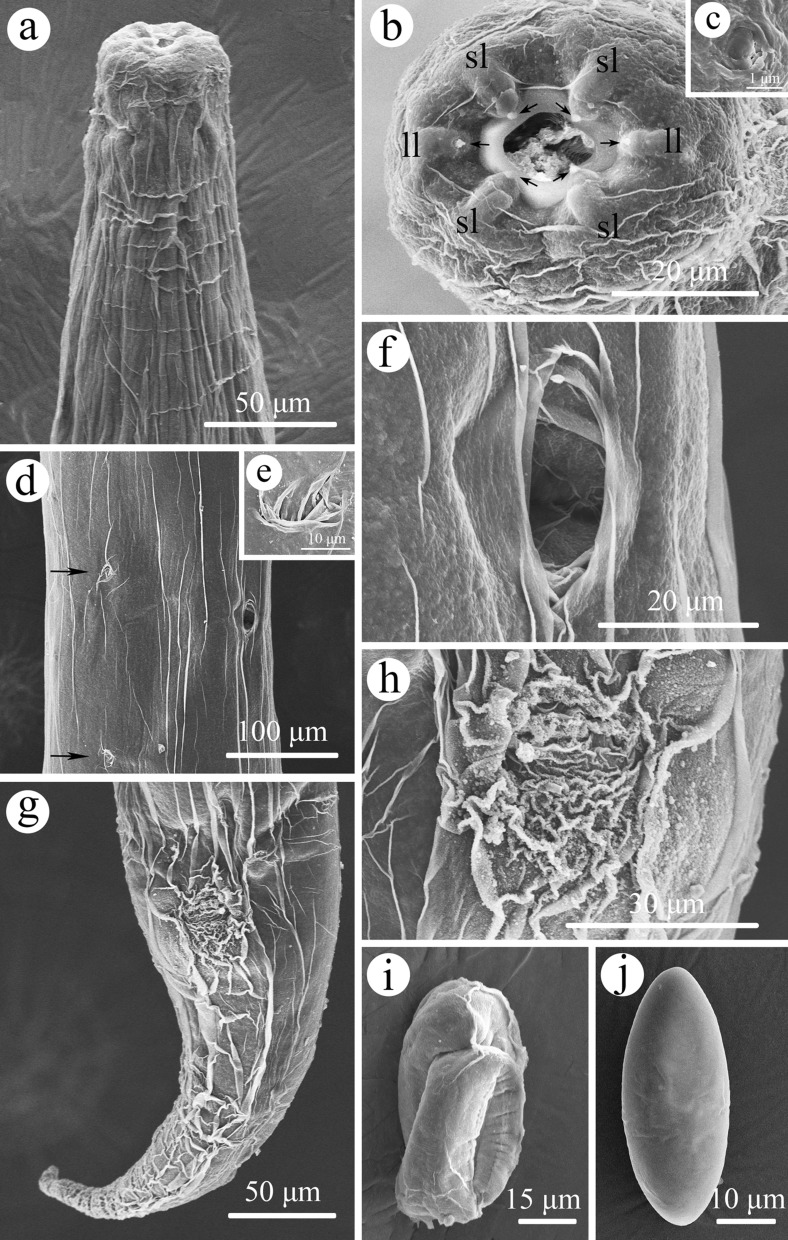
Scanning electron micrographs of *Rhabdias kafunata* from *Bufo gargarizans* in China. **a** anterior part of body, lateral view; **b** cephalic extremity (arrows indicate single papilla on each lip), apical view; **c** magnified image of amphid; **d** mid-body at level of vulva (arrows indicate lateral cuticular pores), sublateral view; **e** magnified image of lateral cuticular pore; **f** magnified image of vulva; **g** posterior end of body, ventral view; **h** magnified image of cloaca; **i** egg with developed larva; and **j** embryonated egg. sl, submedian lip; ll, lateral lip

Small, whitish nematodes, with an conspicuously inflated cuticle along entire body; surface of cuticle has remarkably irregular transverse folds (Figs. 1a–f, 2a–c). Body gradually tapers from mid-region toward anterior and posterior ends. Maximum width at region slightly anterior to vulva (Fig. 1a). Lateral alae absent. Lateral cuticular pores distributed laterally in a longitudinal row along entire body (Figs. 1b, 3d, e). Cephalic extremity rounded. Oral opening simple, more or less square-shaped, surrounded by six small lips (two lateral and four submedian) reduced to elongated elevations (Figs. 2d, 3b); submedian lips located closer to the edge of oral opening than lateral lips, with each lip bearing single papilla (Figs. 2d, 3b). Amphids poroid located at base of lateral lip (Figs. 2d, 3c). Buccal capsule small and cup-like, with well sclerotized walls (Figs. 1c, e, 2a, c). Esophagus club-shaped, with posterior end slightly expanded to esophageal bulb; anterior part of esophagus mostly muscular, posterior part more or less glandular (Figs. 1a, c, 2a). Muscular part slightly dilated at its anterior two-thirds. Uteri didelphic and amphidelphic, typical of *Rhabdias*. Vulval opening in pore, without salient lips (Figs. 1a, d, 2e, 3d, f). Uteri thin-walled, filled with well-developed embryonated or unembryonated eggs. Eggs oval, with smooth thin shells (Figs. 1d, 2e, f, 3i, j). Tail conical, with a pointed tip (Figs. 1a, f, 2b, 3g). Morphometric data of the present specimens and morphometric comparisons of *R. kafunata* among the present material and previous studies are provided in Table 1.

#### Molecular characterization of *Rhabdias kafunata*

Four partial *cox1* sequences of *R. kafunata* obtained here had 655 bp, representing four different genotypes with 0.31–1.22% of nucleotide divergence. Pairwise comparison of the partial *cox1* sequences of *R. kafunata* obtained here with that of *R. kafunata* (LC496790, LC496791) available in GenBank, showed 2.44% of nucleotide divergence. Three partial 12S sequences of *R. kafunata* obtained here had 475 bp, with no nucleotide divergence detected. Pairwise comparison of the partial 12S sequences of *R. kafunata* obtained here with that of *R. kafunata* (LC496792, LC496793) available in GenBank showed 0.63% of nucleotide divergence. There are no 28S or ITS sequences of *R. kafunata* available in GenBank. Four partial 28S sequences of *R. kafunata* obtained here had 553 bp, with no nucleotide divergence detected. Four partial ITS sequences of *R. kafunata* obtained here had 708–711 bp, representing two different genotypes with 0.14% of nucleotide divergence.

### Remarks

Sata et al. [33] originally described *R. kafunata* from *B. gargarizans miyakonis* in Japan, and also provided the *cox1* and 12S sequences for the species. The present specimens collected from *B. gargarizans* in China are almost identical to the original description of *R. kafunata* regarding several features, including body length, morphology of buccal capsule, relative length of esophagus to body length, morphology and length of tail, position of vulva, and size of eggs (Table 1 for details). Moreover, molecular analysis showed only 0.63% and 2.44% of nucleotide divergence in the 12S and *cox1* sequences, respectively, between the present material and the types of *R. kafunata*. Therefore, the present specimens were identified as *R. kafunata*. The detailed morphology of the cephalic structures, vulva, and eggs was observed using SEM for the first time. We also generated 28S and ITS sequences of *R. kafunata* for the first time, which are useful for the phylogeny and molecular diagnosis of this species. *Rhabdias kafunata* is then reported in China for the first time, and represents the ninth species of *Rhabdias* recorded in the country.

#### ***Rhabdias bufonis*** (Schrank, 1788; Figs. 4–6; Table 2)

**Fig. 4:**
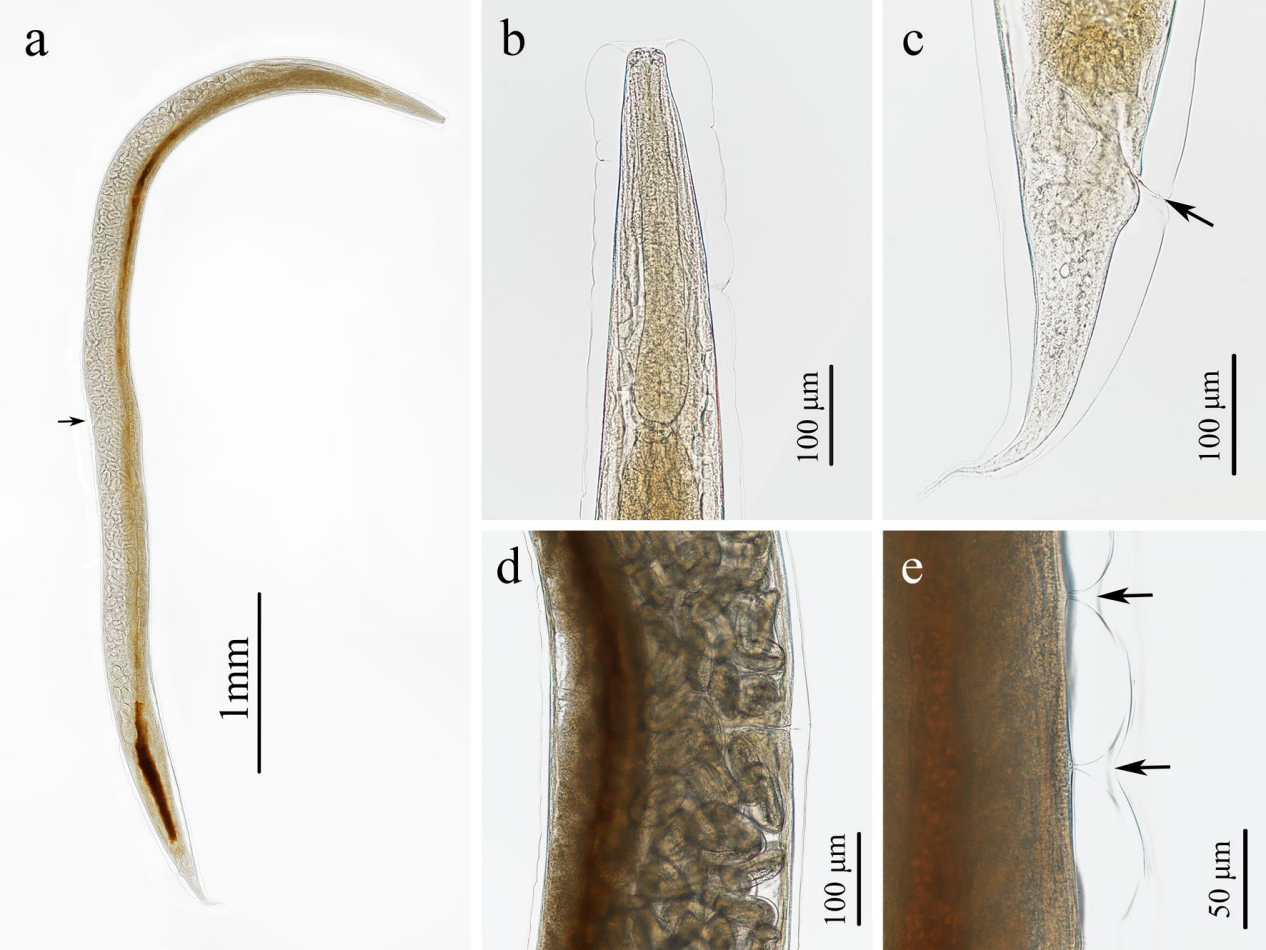
Photomicrographs of *Rhabdias bufonis* from *Bufo gargarizans* in China. **a** Entire body (arrow indicates vulva), lateral view; **b** anterior part of body, lateral view; **c** posterior part of body (arrow indicates cloaca), lateral view; **d** region of vulva, lateral view; and **e** lateral cuticular pores (arrow), lateral view

**Table 2:** Morphometric comparisons of *Rhabdias bufonis*

Characteristics	Present study	Hartwich (1972)	Wang et al. (1992)	Kuzmin (2013)
	Female	Female	Female	Female
BL (mm)	6.10–7.49	6.44–18.4	4.80–7.52	3.88–13.0
BMW	242–386	259–558	320–336	99–340
SBC	10.0–12.5 × 7.50–10.0	–	–	10.0–12.0 × 8.00–12.0
EL	391–444	441–576	352–400	324–589
AW	25.0–30.0	–	36.0–48.0	22.0–42.0
DW	35.0–42.5	–	–	28.0–45.0
GW	27.5–42.5	–	–	26.0–45.0
BW	52.5–67.5	–	64.0–68.0	45.0–77.0
NRC	180–210	–	160–176	133–241
EPC	217–256	–	–	–
VC (mm)	3.12–3.98	3.37–10.1	2.24–3.52	2.20–7.00
TL	242–353	200–359	280–320	192–498
SE	34–58 × 68–106	–	52–54 × 98–105	47–64 × 100–119
EL/BL (%)	5.42–6.65	2.81–6.85	–	4.10–9.90
VC/BL (%)	50.2–54.3	45.5–58.9	–	50.7–58.7
TL/BL (%)	3.35–4.71	1.65–3.11	–	2.40–5.50
Host	*Bufo gargarizans*	*Bufo bufo*	*Duttaphrynus melanostictus*	*Rana temporaria*, *R. arvalis*
Country	China	Germany	China	Ukraine

Measurements are given in micrometers unless otherwise stated. BL, length of body; BMW, maximum width of body; SBC, size of buccal capsule; EL, length of entire esophagus; AW, width of anterior part of esophagus; DW, width of esophageal dilatation; GW, width of middle glandular part of esophagus; BW, width of posterior end of esophagus; NRC, distance of nerve-ring to cephalic end; EPC, distance of excretory pore to cephalic end; VC, distance of vulva to cephalic end; TL, length of tail; SE, well-developed, embryonated eggs

*Present host*: Asiatic toad *Bufo gargarizans* Cantor (Amphibia: Anura).

*Present locality*: Shijiazhuang, Hebei Province, China.

*Site in host*: Lung.

*Prevalence and intensity of infection*: 45 of 50 (90.0%) toads examined, and infected with an intensity of 4–121 (mean 45.0) nematodes per infected host (a total of 1161 female nematode specimens collected).

*GenBank accession*: OR690323–OR690327 (28S), OR690329–OR690333 (ITS), OR690336–OR690340 (*cox1*), OR711500–OR711503 (12S), OR725306 (mitochondrial genome).

#### Morphological description

**Fig. 5:**
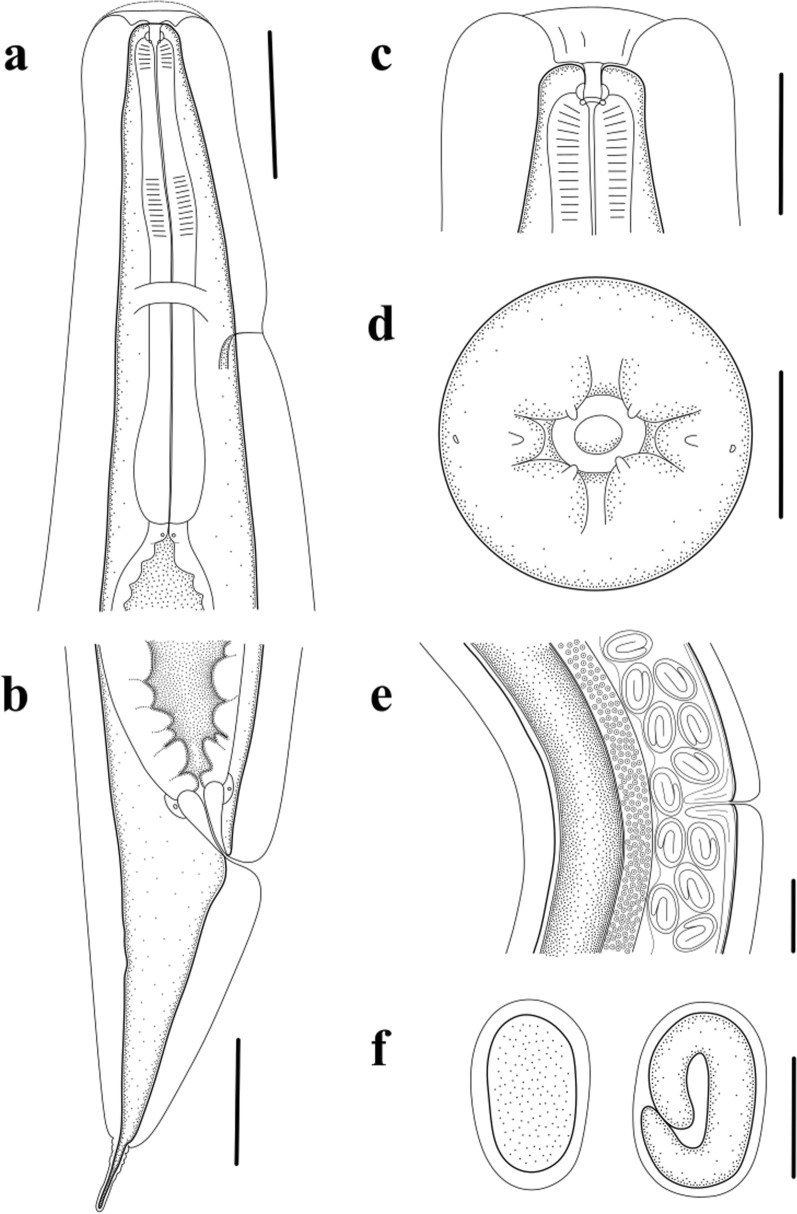
Line drawings of *Rhabdias bufonis* from *Bufo gargarizans* in China. **a** anterior part of body, lateral view; **b** tail, lateral view; **c** cephalic extremity, lateral view; **d** cephalic extremity, apical view; **e** region of vulva, lateral view; **f** eggs. Scale bars: **a**, **b**, **e**, 100 μm; **c**, **f**, 50 μm; **d**, 10 μm

**Fig. 6:**
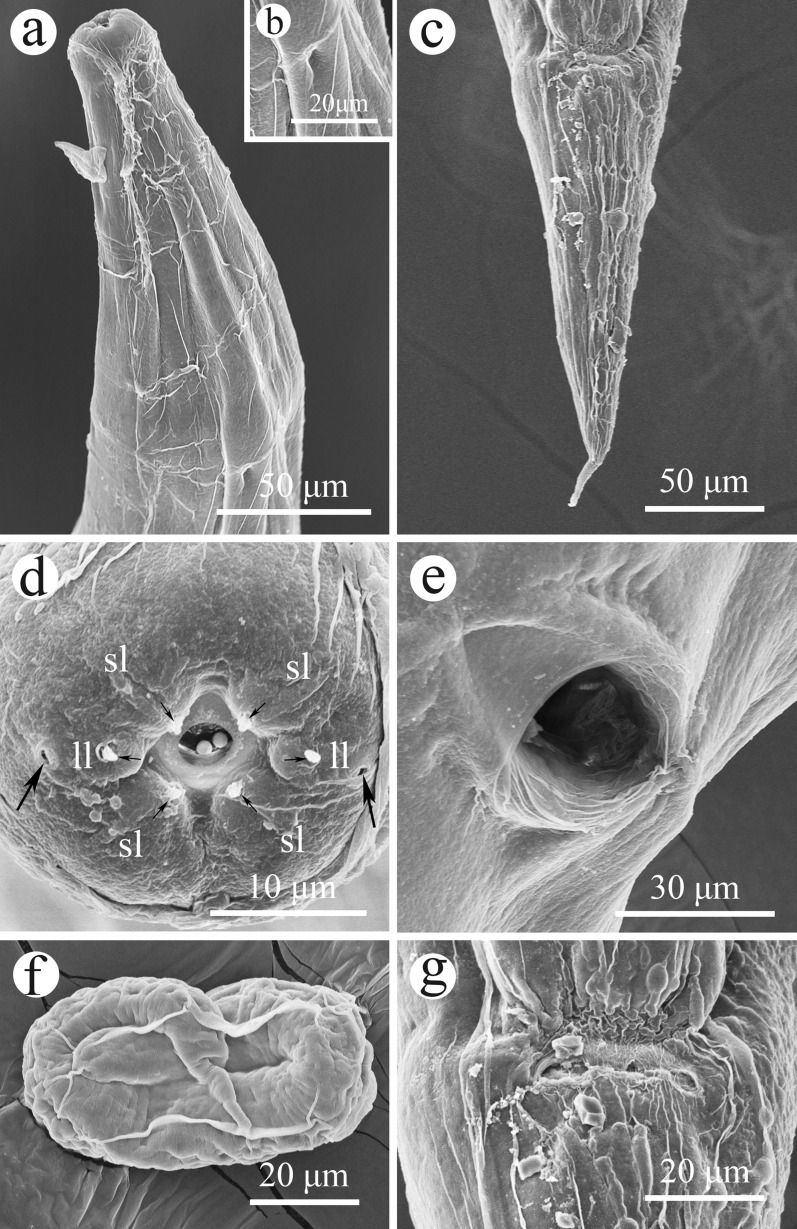
Scanning electron micrographs of *Rhabdias bufonis* from *Bufo gargarizans* in China. **a** anterior part of body, ventral view; **b** magnified image of excretory pore; **c** tail, ventral view; **d** cephalic extremity (arrows indicate amphids and single papilla on each lip), apical view; **e** magnified image of vulva; **f** egg with developed larva; and **g** magnified image of cloaca. sl, submedian lip; ll, lateral lip

Small whitish nematodes with a conspicuously inflated cuticle along entire body. Surface of cuticle slightly irregular transverse folds (Figs. 4a–c, e, 5a–c). Body gradually tapers from mid-region toward anterior and posterior ends. Maximum width at the level of vulva (Fig. 4a). Lateral alae absent. Lateral cuticular pores distributed laterally in a longitudinal row along entire body (Fig. 4e). Cephalic extremity rounded. Oral opening simple, more or less rounded, surrounded by six small lips (two lateral and four submedian; Figs. 5d, 6d); submedian lips located closer to edge of oral opening than lateral lips; each lip bears single papilla (Figs. 5d, 6d). Amphid poroid located at base of lateral lip (Figs. 5d, 6d). Buccal capsule small and funnel-shaped, with well-sclerotized walls (Fig. 5a, c). Esophagus club-shaped, a postetrior end slightly expanded; anterior part of esophagus mostly muscular, posterior part nearly glandular (Figs. 4a, b, 5a). Muscular part slightly dilated at its anterior one-third (Figs. 4a, b, 5a). Uteri didelphic and amphidelphic, typical of *Rhabdias*. Vulval opening in the pore, without salient lips (Figs. 4a, d, 5e, 6e). Uteri thin-walled, filled with well-developed embryonated or unembryonated eggs (Figs. 4a, d, 5e). Eggs oval and thin-shelled, with smooth surfaces (Figs. 4a, d, 5e, f, 6f). Tail conical, with a pointed tip (Figs. 4a, c, 5b, 6c). Morphometric data of the present specimens and morphometric comparisons of *R. bufonis* among the present material and previous studies are provided in Table 2.

#### Molecular characterization of *Rhabdias bufonis*

Five partial 28S sequences of *R. bufonis* obtained here had 553–559 bp, with no nucleotide divergence detected. Pairwise comparison of these partial 28S sequences of *R. bufonis* with that of *R. bufonis* (KF999593) and *R. cf. bufonis* (KF999606, KF999609) available in GenBank, showed 0–0.37% of nucleotide divergence. Five partial ITS sequences of *R. bufonis* obtained here had 701–709 bp, with no nucleotide divergence detected. Pairwise comparison of these partial ITS sequences of *R. bufonis* with that of *R. bufonis* (KF999593) and *R. cf. bufonis* (KF999606, KF999609) available in GenBank, showed 0.15% of nucleotide divergence. Four partial 12S sequences of *R. bufonis* obtained here had 474 bp, with no nucleotide divergence detected. Pairwise comparison of these partial 12S sequences of *R. bufonis* obtained here with that of *R. bufonis* (MK680940) available in GenBank showed 0.86% of nucleotide divergence. Five partial *cox1* sequences of *R. bufonis* obtained here had 655 bp, representing three different genotypes with 0.31–1.07% of nucleotide divergence. Pairwise comparison of these partial *cox1* sequences of *R. bufonis* with that of *R. bufonis* (MK681425) available in GenBank showed 6.24% of nucleotide divergence.

### Remarks

*Rhabdais bufonis* has been reported from various frogs and toads worldwide [4, 34–39], in which some of these reports are based on redescription of the species [4, 34, 37]. However, some specific identifications of *R. bufonis* are questionable [4]. The present specimens collected from *B. gargarizans* in China are largely similar to the redescriptions of *R. bufonis*, regarding several features, including body length, morphology and lengths of esophagus and tail, position of vulva, and size of eggs (Table 2 for details). Therefore, we considered the present material conspecific to *R. bufonis*. Moreover, genetic sequences showed only 0–0.37%, 0.15%, and 0.86% of nucleotide divergence in the 28S, ITS, and 12S data of *R. bufonis* available in GenBank. The detailed morphology of the cephalic structures, vulva and eggs of *R. bufonis* was observed using SEM for the first time. The general morphology of *R. bufonis* is very similar to that of *R. nipponica*, but the body length and egg size of *R. nipponica* are distinctly smaller than that of *R. bufonis* (body length 6.10–7.49 mm and egg width 0.034–0.058 mm in *R. bufonis* versus body length 4.12–5.52 mm and egg width not exceeding 0.05 mm in *R. nipponica*) [4, 40]. Additionally, *R. nipponica* has been recorded only infecting frogs and never toads.

Marcaida et al. [41] reported the *cox1* sequences of *R. nipponica* collected from different hosts in Japan, which displayed 0.13% (LC671278 versus LC671277) to 9.65% (LC671283 versus LC671275) of nucleotide divergence between different samples. This result seems to suggest that *R. nipponica* could possibly be a complex comprising several sibling species. The similar situation maybe also occurs in *R. bufonis,* because molecular analysis of the *cox1* and 12S data of *R. bufonis* between our material and the specimens collected from *Rana temporaria* in Ukraine [42] showed a low level of nucleotide divergence in 12S region (0.86%), but a high level of nucleotide divergence in the *cox1* region (6.24%). Moreover, the specimens of *R. bufonis* collected from Europe (Germany and Ukraine) showed remarkable morphological variability in body length and relative length of entire esophagus and tail to body length (see Table 2 for details), which may be the result of the different infection intensity, localities, or host species, but may also indicate that the European specimens of *R. bufonis* possibly represent a species complex. Additionally, the level of nucleotide divergence between *R. bufonis* and *R. engelbrechti* are 0.57–1.13% in ITS, 0.56% in 12S, and 4.36–6.63% in the *cox1* region, but *R. engelbrechti* with divided buccal capsule is different from *R. bufonis* [43]. A more rigorous molecular analysis with samples of *R. bufonis* collected from different localities and hosts worldwide (especially from Europe) is required to solve the taxonomic status of this species.

### General characterization of complete mitogenomes of *Rhabdias kafunata* and *R. bufonis*

**Fig. 7:**
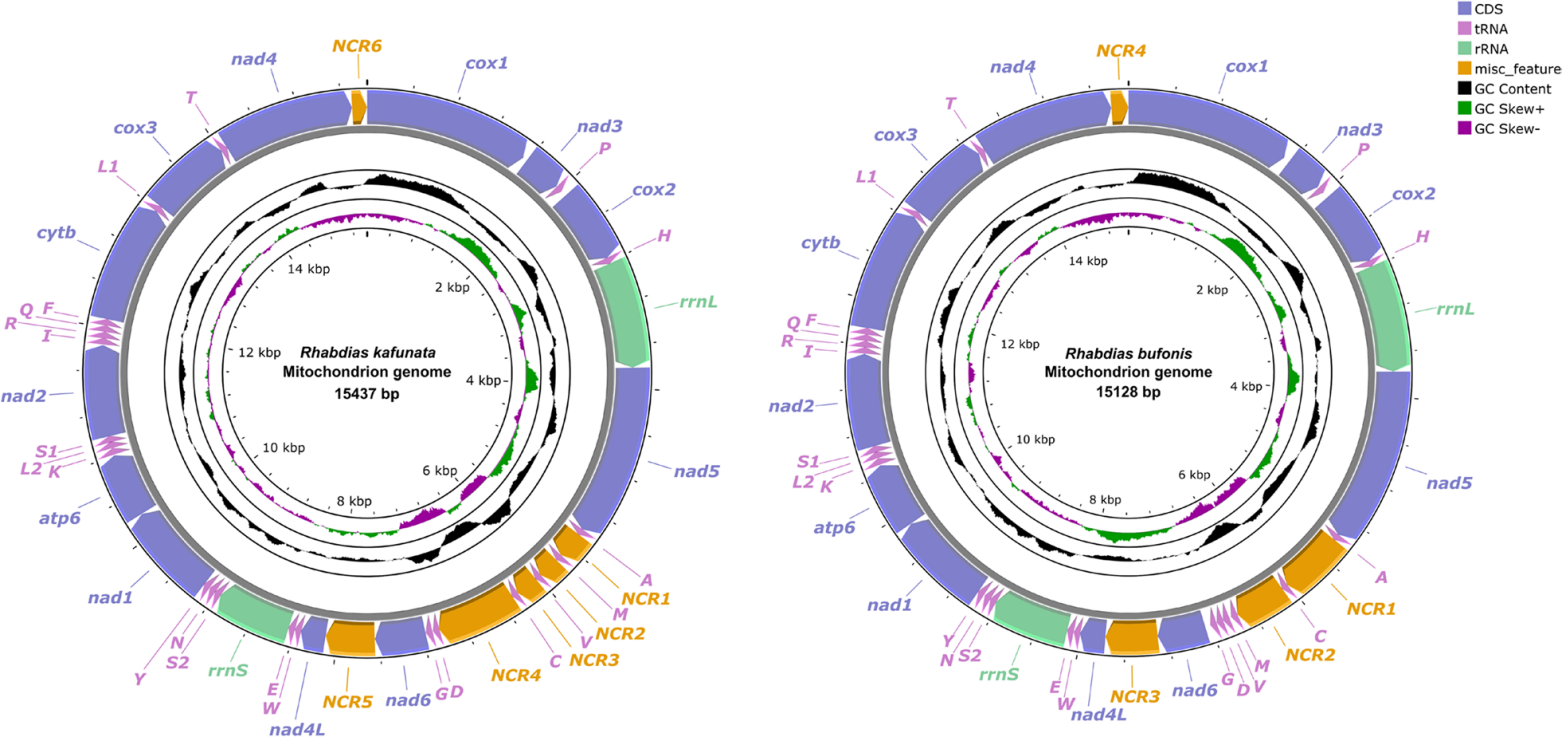
Gene maps of the mitochondrial genomes of *Rhabdias kafunata* and *Rhabdias bufonis*. NCR, non‑coding region; PCG, protein‑coding gene; rRNA, ribosomal RNA; tRNA, transfer RNA

The mitogenome of *R. kafunata* and *R. bufonis* had 15,437 bp and 15,128 bp, respectively, both containing 36 genes, including 12 PCGs (missing *atp*8; *cox1*–*3*, *cyt*b, *nad*1–6, *nad*4L and *atp*6), 22 tRNA genes and 2 rRNA genes (*rrn*L and *rrn*S; Fig. 7). All genes were transcribed from the same DNA strand. There were six non-coding regions in the mitogenome of *R. kafunata* (NCR1 is 253 bp, between *tRNA-Ala* and *tRNA-Met*; NCR2 is 204 bp, between *tRNA-Met* and *tRNA-Val*; NCR3 is 214 bp, between *tRNA-Val* and *tRNA-Cys*; NCR4 is 747 bp, between *tRNA-Cys* and *tRNA-Asp*; NCR5 is 456 bp, between *nad*6 and *nad*4L; and NCR6 is 139 bp, located behind the *nad*4 gene). In contrast, there were four non-coding regions in the mitogenome of *R. bufonis* (NCR1 is 596 bp, between *tRNA-Ala* and *tRNA-Cys*; NCR2 is 471 bp, between *tRNA-Cys* and *tRNA-Trp*, NCR3 is 476 bp, between *nad*6 and *nad*4L; and NCR4 is 156 bp, located behind the *nad*4 gene; Fig. 7). The nucleotide contents of mitogenomes of *R. kafunata* and *R. bufonis* are provided in Additional file 5: Table S5, Additional file 6: Table S6. The overall A + T contents in the mitogenomes of *R. kafunata* and *R. bufonis* were 75.8% and 76.7%, respectively, both showing a strong nucleotide compositional bias toward A + T (Table 3).

**Table 3:** Base composition and skewness of *Rhabdias kafunata* and *Rhabdias bufonis*

Location/species	Total	A (%)	T (%)	C (%)	G (%)	A + T (%)	AT skew	GC skew
*Rhabdias kafunata*								
Complete mitochondrial genome	15,437	28.7	47.2	7.8	16.5	75.8	−0.24	0.36
Protein coding genes (PCGs)	10,327	25.7	49.8	7.6	16.9	75.5	−0.32	0.38
Codon position								
First codon	3442	30.4	41.5	7.1	21.0	71.9	−0.15	0.49
Second codon	3442	19.2	51.1	12.8	16.9	70.3	−0.45	0.14
Third codon	3442	27.6	56.8	2.9	12.7	84.4	−0.35	0.62
tRNAs	1238	35.1	39.7	8.2	17.0	74.8	−0.06	0.35
rRNAs	1696	35.7	41.3	7.4	15.6	77.0	−0.07	0.36
*rrn*L	993	35.6	43.7	6.1	14.6	79.3	−0.10	0.41
*rrn*S	703	35.9	38.0	9.1	17.1	73.8	−0.03	0.30
Non-coding region 1	253	36.4	45.8	5.1	12.6	82.2	−0.11	0.42
Non-coding region 2	204	28.9	25.5	21.6	24.0	54.4	0.06	0.05
Non-coding region 3	214	38.3	45.8	3.7	12.1	84.1	−0.09	0.53
Non-coding region 4	747	37.9	39.2	9.8	13.1	77.1	−0.02	0.14
Non-coding region 5	456	27.2	51.5	6.8	14.5	78.7	−0.31	0.36
Non-coding region 6	139	25.2	46.0	10.1	18.7	71.2	−0.29	0.30
*Rhabdias bufonis*								
Whole mitochondrial genome	15,128	28.5	48.2	7.4	15.9	76.7	−0.26	0.36
Protein coding genes (PCGs)	10,246	25.7	50.5	7.4	16.3	76.2	−0.32	0.37
Codon position								
First codon	3415	29.9	41.9	7.1	21.2	71.8	−0.17	0.50
Second codon	3415	19.2	51.1	12.9	16.8	70.2	−0.45	0.13
Third codon	3415	28.1	58.5	2.3	11.0	86.6	−0.35	0.65
tRNAs	1241	34.1	40.1	8.5	17.2	74.2	−0.08	0.34
rRNAs	1686	35.0	41.3	7.7	16.0	76.3	−0.08	0.35
*rrn*L	984	34.1	43.9	6.7	15.2	78.0	−0.13	0.39
*rrn*S	702	36.2	37.7	9.0	17.1	73.9	−0.02	0.31
Non-coding region 1	596	31.0	45.6	9.2	14.1	76.7	−0.19	0.21
Non-coding region 2	471	44.8	45.2	4.5	5.5	90.0	0.00	0.10
Non-coding region 3	476	27.7	51.9	4.8	15.5	79.6	−0.30	0.53
Non-coding region 4	156	29.5	47.4	8.3	14.7	76.9	−0.23	0.28

The 12 PCGs of the mitogenomes of *R. kafunata* and *R. bufonis* had 10,327 bp and 10,246 bp, respectively, (excluding termination codons) and ranged in size from 234 bp (*nad*4L) to 1590 bp (*nad*5), which encoded 3442 and 3415 amino acids, respectively (Additional file 5: Table S5, Additional file 6: Table S6). Among the 12 PCGs of *R. kafunata*, five genes (*nad*1, *nad*2, *cox2*, *cox3* and *nad*4) used TTG as the start codon, followed by ATN for seven PCGs (ATA: *cox3* and *cyt*b; ATG: *cox1*; and ATT: *nad*4L, *nad*5, *nad*6, and *atp*6). TAA was the most commonly used termination codon (*cox1*, *cox2*, *cox3*, *nad*1, *nad*3, *nad*4, *nad*4L, *nad*5, *nad*6, and *atp*6). *nad*2 used TAG, while the incomplete termination codons T was inferred only for the *cyt*b genes (Additional file 5: Table S5). Among the 12 PCGs of *R. bufonis*, five genes (*nad*1, *nad*2, *cox*3, *cyt*b, and *nad*4) used TTG as the start codon, whereas four genes (*cox1*, *nad*5, *nad*4L, and *atp*6) used ATT, and ATA was used by *nad*3, *cox2*, and *nad*6. TAA was the most commonly used termination codon (*cox1*, *cox3*, *nad*1, *nad*2, *nad*3, *nad*4, *nad*4L, *nad*5, *nad*6, and *atp*6), *cox2* used TAG, and the incomplete termination codon T was inferred only for the *cyt*b genes (Additional file 6: Table S6). The component and usages of codons in the mitogenomes of *R. kafunata* and *R. bufonis* were shown in Additional file 7: Fig. S1. The length of 22 tRNAs and their anticodons secondary structures of *R. kafunata* and *R. bufonis* were provided (Additional file 8: Fig. S2, Additional file 9: Fig. S3, Additional file 5: Table S5, Additional file 6: Table S6).

**Fig. 8:**
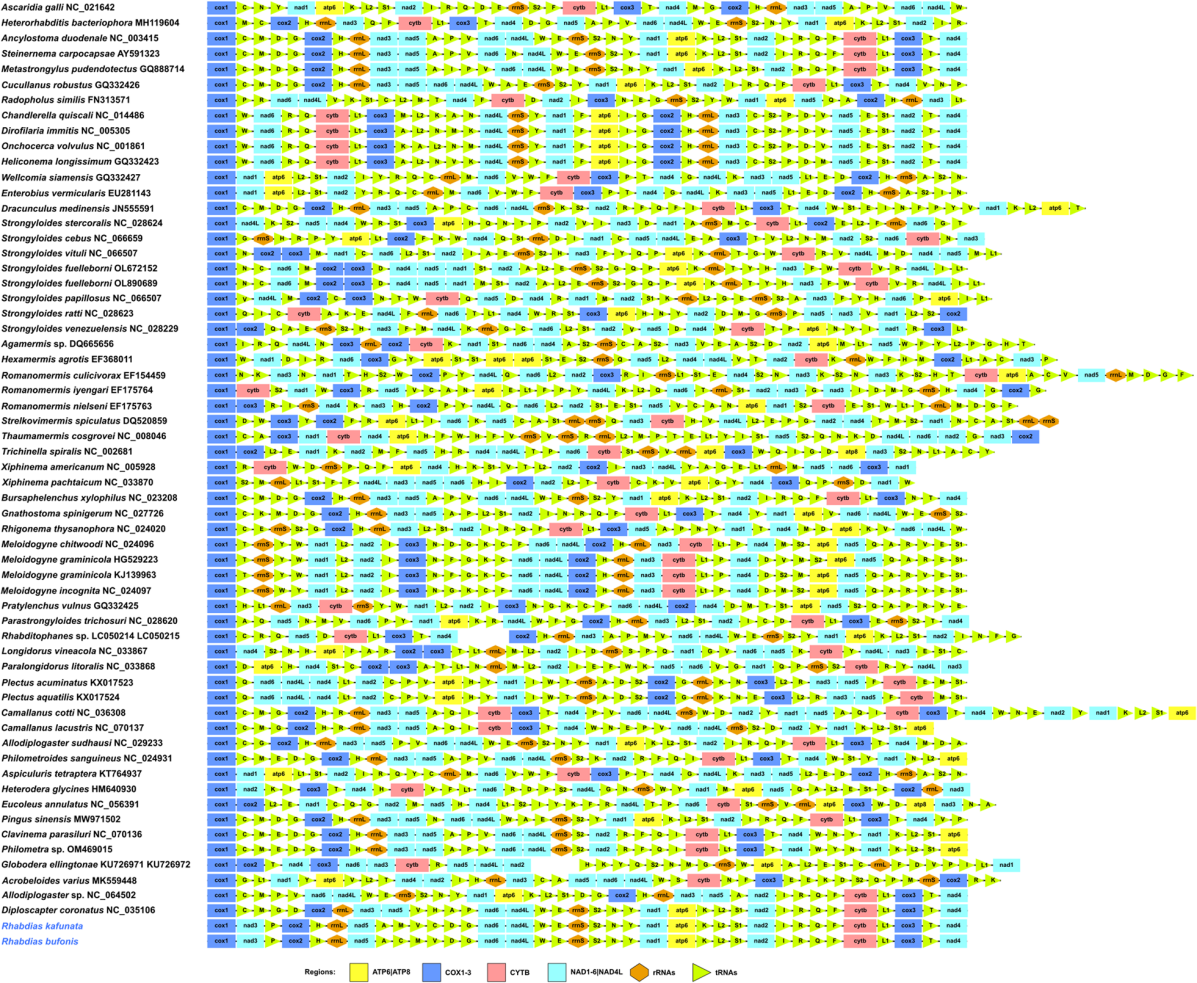
Linearized representation of the nematode mitochondrial gene arrangement of nematodes. The non-coding regions are not indicated

The 36-gene arrangement in the mitogenomes of *R. kafunata* and *R. bufonis* differs from any of the arrangement types reported so far for Nematoda (Fig. 8). The arrangement in *R. kafunata* is in the following order: *cox1*, *nad*3, *tRNA-Pro*, *cox2*, *tRNA-His*, *rrn*L, *nad*5, *tRNA-Ala*, *tRNA-Met*, *tRNA-Val*, *tRNA-Cys*, *tRNA-Asp*, *tRNA-Gly*, *nad*6, *nad*4L, *tRNA-Trp*, *tRNA-Glu*, *rrn*S, *tRNA-Ser*2, *tRNA-Asn*, *tRNA-Tyr*, *nad*1, *atp*6, *tRNA-Lys*, *tRNA-Leu*2, *tRNA-Ser*1, *nad*2, *tRNA-Ile*, *tRNA-Arg*, *tRNA-Gln*, *tRNA-Phe*, *cyt*b, *tRNA-Leu*1, *cox3*, *tRNA-Thr*, and *nad*4 (Figs. 7, 8). The arrangement in *R. bufonis* is in the following order: *cox1*, *nad*3, *tRNA-Pro*, *cox2*, *tRNA-His*, *rrn*L, *nad*5, *tRNA-Ala*, *tRNA-Cys*, *tRNA-Met*, *tRNA-Val*, *tRNA-Asp*, *tRNA-Gly*, *nad*6, *nad*4L, *tRNA-Trp*, *tRNA-Glu*, *rrn*S, *tRNA-Ser*2, *tRNA-Asn*, *tRNA-Tyr*, *nad*1, *atp*6, *tRNA-Lys*, *tRNA-Leu*2, *tRNA-Ser*1, *nad*2, *tRNA-Ile*, *tRNA-Arg*, *tRNA-Gln*, *tRNA-Phe*, *cyt*b, *tRNA-Leu*1, *cox*3, *tRNA-Thr*, and *nad*4 (Figs. 7, 8).

### Phylogenetic analyses

**Fig. 9:**
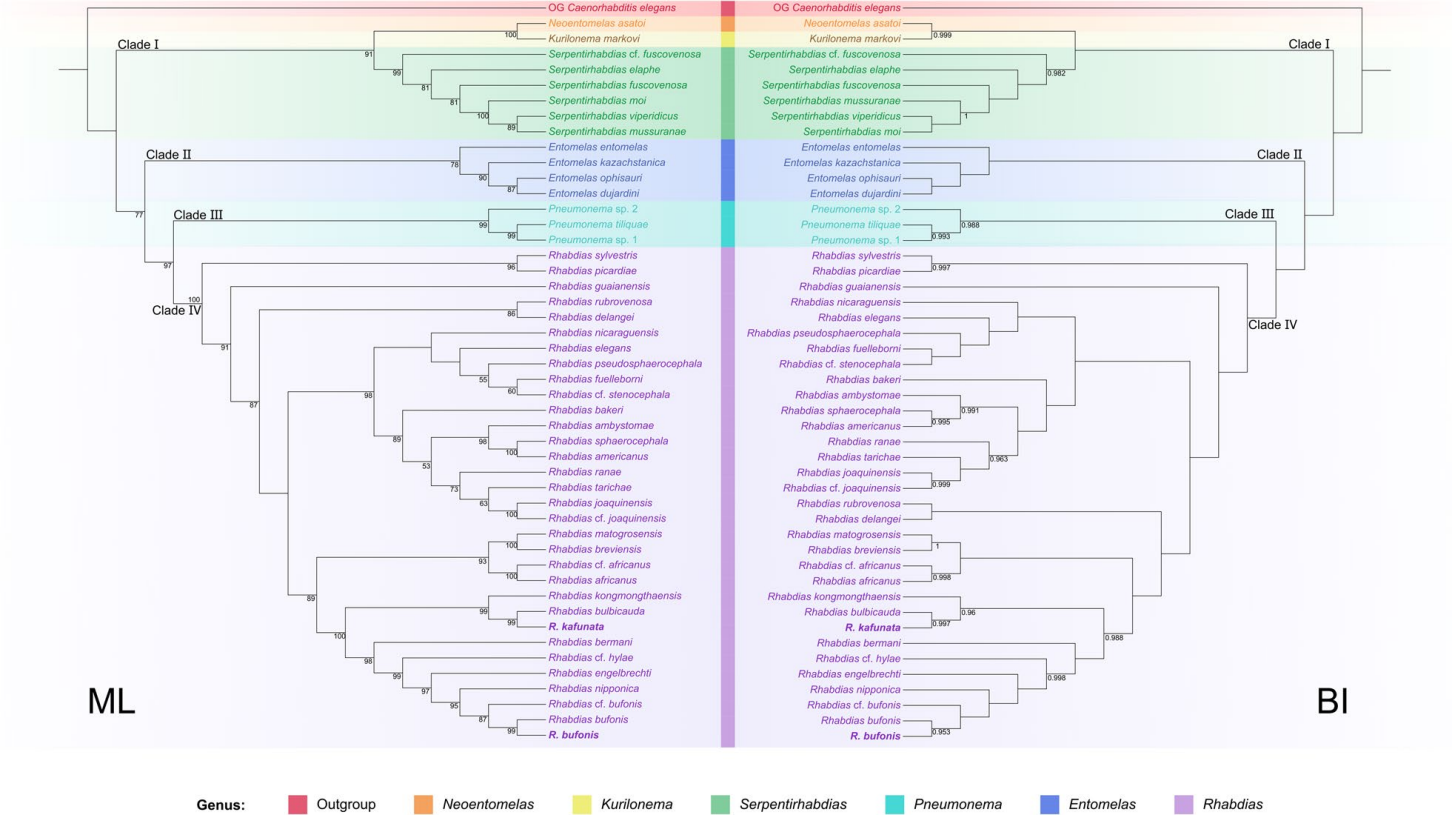
Maximum likelihood (ML) inference and Bayesian inference (BI) based on the ITS + 28S sequence data showing the phylogenetic relationships of representatives of Rhabdiasidae. *Caenorhabditis elegans* Dougherty, 1953 (Rhabditida: Rhabditidae) was chosen as the out-group. Bootstrap values ≥ 50 and Bayesian posterior probabilities values ≥ 0.95 are shown in the phylogenetic trees. Bold indicates *Rhabdias kafunata* and *R. bufonis*

Phylogenetic trees constructed on the basis of the ITS + 28S sequence data using ML and BI had almost identical topologies (Fig. 9). The representatives of Rhabdiasidae were divided into four large clades (clade I, II, III, and IV). Clade I included species of *Neoentomelas*, *Kurilonema*, and *Serpentirhabdias*. Among them, *Neoentomelas* and *Kurilonema* clustered together, being sister to *Serpentirhabdias*. Clade II contained representatives of *Entomelas*. Clade III consisted of species of *Pneumonema*. Clade IV included the representatives of *Rhabdias*. In the clade IV, *R. kafunata* was sister to *R. bulbicauda,*, and *R. bufonis* was sister to *R. nipponica*.

**Fig. 10:**
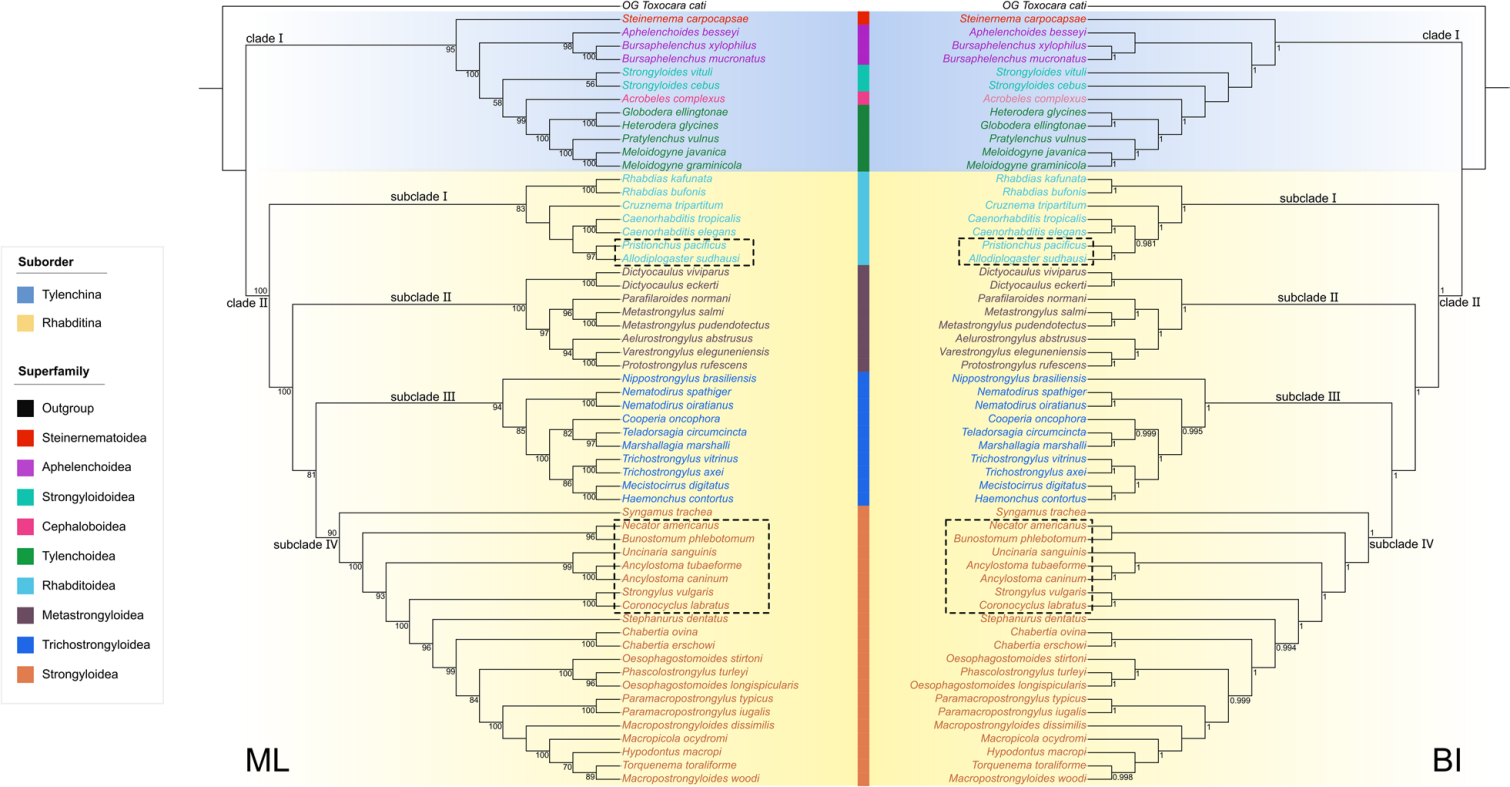
Maximum likelihood (ML) inference based on the amino acid sequences of 12 protein-coding genes (PCGs) of mitochondrial genomes showing the phylogenetic relationships of representatives of Tylenchina and Rhabditina. *Toxocara cati* (Ascaridida: Toxocaridae) was chosen as the out-group. Bootstrap values ≥ 50 and Bayesian posterior probabilities values ≥ 0.95 are shown in the phylogenetic trees. Dotted boxes indicated the present phylogenetic results did not supported the validity of the family Diplogasteridae and the superfamily Ancylostomatoidea in the traditional classification

Phylogenetic results based on the amino acid sequences of 12 PCGs of mitogenomes using ML and BI had almost identical topologies (Fig. 10). The representatives of the Rhabditida were divided into two large monophyletic clades, representing the suborders Tylenchina (clade I) and Rhabditina (clade II), respectively. In clade I (Tylenchina), the superfamily Steinernematoidea (including only *Steinernema carpocapsae*) was basal. The representative of the superfamily Cephaloboidea (including only *Acrobeles complexus*) formed a sister lineage to the superfamily Tylenchoidea.

In the clade II, representatives of Rhabditina were divided into four subclades (I, II, III, and IV). In the subclade I (representing the superfamilies Rhabditoidea + Diplogasteroidea), species of the Diplogasteroidea/Diplogasteridae (including *Pristionchus pacificus* and *Allodiplogaster sudhausi*) clustered with representatives of Rhabditidae (including *Cruznema tripartitum*, *Caenorhabditis elegans* and *C. tropicalis*), all of which were sister to the family Rhabdiasidae (including *R. kafunata* and *R. bufonis*). In the subclade II (representing the superfamily Metastrongyloidea), *Parafilaroides normani* (Filaroididae) nested with the representatives of Metastrongylidae. In the subclade III (representing the superfamily Trichostrongyloidea), the family Heligmonellidae (including only *Nippostrongylus brasiliensis*) was basal, and the family Molineidae (including *Nematodirus oiratianus* and *N. spathiger*) was sister to the representatives of Trichostrongylidae. In the subclade IV (representing the superfamily Strongyloidea), the family Syngamidae (including only *Syngamus trachea*) was basal. The family Ancylostomatidae (including *Necator americanus*, *Uncinaria sanguinis*, *Bunostomum phlebotomum*, *Ancylostoma tubaeforme*, and *A. caninum*) was not monophyletic. Phylogenetic results also indicated the family Strongylidae as a monophyletic lineage.

## Discussion

In the present study, 50 individuals of *B. gargarizans* were examined, with 8 individuals co-infected by *R. kafunata* and *R. bufonis*. *Rhabdias kafunata* can be easily distinguished from *R. bufonis* by its distinctly longer body and esophagus and much larger buccal capsule (body length 13.3–17.1 mm and esophageal length 0.81–0.90 mm and size of buccal capsule 0.020–0.025 × 0.028–0.038 mm in *R. kafunata* versus body length 6.10–7.49 mm and esophageal length 0.39–0.44 mm and size of buccal capsule 0.01–0.013 × 0.008–0.010 mm in *R. bufonis*).

In recent decades, a large number of mitogenomes of nematodes have been published, which contributes to the knowledge regarding their phylogeny and evolutionary history [7, 8, 44, 45]. However, several families still have all representatives with unknown mitogenomic data. In the present study, the complete mitochondrial genomes of *R. kafunata* and *R. bufonis* were provided for the first time. The size of the complete mitogenomes of *R. kafunata* (15,437 bp) and *R. bufonis* (15,128 bp) were shown to be larger than that of other species belonging to Rhabditina (*Dictyocaulus viviparus* 13,310 bp to *Syngamus trachea* 14,647 bp), with exception to that of *Mecistocirrus digitatus* (15,221 bp). The composition of the complete mitogenomes of *R. kafunata* and *R. bufonis* [including 12 PCGs (missing *atp*8), 22 tRNA genes, and 2 rRNA genes] was typical of most nematode mitogenomes, except for those of *Trichinella* spp. and *Trichuris* spp., which have the *atp*8 gene [46–50]. A recent study claimed to sequence the mitogenome of *R. kafunata* (OP605735) [51]. However, this study did not provide any information on the species identification of their specimens. Pairwise comparison of the partial *cox1* sequences of *R. kafunata* obtained here and available in Genbank (LC496790, LC496791) with that of the mitogenome of *R. kafunata* (OP605735) showed 4.43–4.89% of nucleotide divergence, which is distinctly higher than the level of intraspecific genetic variation of *R. kafunata* (0.31–2.44%) and is similar to the level of interspecific genetic variation of some *Rhabdias* spp. (i.e., the level of interspecific genetic variation of *R. bufonis* and *R. engelbrechti* is 4.36–6.63% in the*cox1* region). Moreover, the size and gene rearrangement of the mitogenome of *R. kafunata* in Li et al. [51] are also different from the present study. Consequently, we considered the specimens identified as *R. kafunata* by Li et al. [51] to be not conspecific with *R. kafunata*.

Kim et al. [52] indicated that mitogenomes of the Enoplea display much higher level of gene rearrangement than that in the members of Chromadorea. Currently, there are 60 types of mitochondrial gene arrangements found in Nematoda, according to the differences in the position of the 12 or 13 PCGs, 22 tRNA genes, and 2 rRNA genes (Fig. 8). In comparison to these types of gene arrangement, one PCG (*nad*4) and several tRNA genes (*tRNA-Ala*, *tRNA-Met*, *tRNA-Cys*, *tRNA-Asp*, and *tRNA-Gly*) rearrangement events occurred in the two rhabdiasid species. Additionally, the present study also revealed the presence of tRNA translocations (*tRNA-Met*, *tRNA-Val*/*tRNA-Cys*), when comparing the mithocondrial genomes of *R. kafunata* and *R. bufonis*. Such tRNA translocations are much more common than protein coding gene or rRNA gene translocations in Nematoda, especially in closely related congeneric species [7]. However, the mitochondrial gene arrangement in *R. kafunata* and *R. bufonis* was consistently different from all of the known mitogenomes of nematodes.

Currently, the phylogenetic knowledge of the family Rhabdiasidae is limited. The present phylogenetic analyses based on ITS + 28S sequences included the most comprehensive taxa sampling of Rhabdiasidae so far. Within this family, the validity of the genus *Kurilonema* remains under debate. Baker [2] considered *Kurilonema* to be a synonym of *Entomelas*. However, such a proposal was rejected in the subsequent studies [4, 53, 54]. The present results supported the validity of *Kurilonema*, and indicated that this genus is sister to *Neoentomelas* with strong support in both ML and BI analyses (BS = 100, BPP = 0.999), which are consistent with the morphological hypothesis [53, 54]. The close affinity between *Kurilonema* and *Neoentomelas* can be depicted through the similar shape of buccal capsule, and in the fact that members of these genera are parasites specific to skinks (Scincidae) in eastern Asia [3, 54].

The present results supported the monophyly of *Entomelas*, *Pneumonema*, *Serpentirhabdias*, and *Rhabdias*, but only the branches of *Serpentirhabdias* spp. and *Entomelas* spp. have strong support in both ML and BI analyses (BS = 99, BPP = 0.982/0.988). Among them, *Pneumonema* was sister to *Rhabdias*, similar to the observations in previous studies [5, 55–58]. In the genus *Rhabdias*, *R. kafunata* was closely related to *R. bulbicauda* with strong support in both ML and BI analyses (BS = 99, BPP = 0.997), but *R. bufonis* was closely related to *R. nipponica* with strong support only in ML tree (BS = 95). Baker [59] presumed that the ancestors of the Rhabdiasidae initially parasitized amphibians and colonized reptiles afterwards. However, the present phylogeny indicated that the ancestors of rhabdiasids initially infected reptiles prior to amphibians.

The present study represented the first attempt to resolve the systematic position of Rhabdiasidae using phylogenetic analyses based on mitogenomic data. In this sense, the present results supported the monophyly of Tylenchina and Rhabditina, and indicated that Rhabdiasidae is closely related to Rhabditidae in the superfamily Rhabditoidea of Rhabditina. Therefore, these results are partially consistent with the traditional classification of Anderson & Bain [21], but contrary to other studies [10, 23]. Some previous studies considered the families Rhabdiasidae and Strongyloididae to have a close relationship [10, 21, 23], but the present mitogenomic phylogenies rejected this hypothesis and placed Rhabdiasidae and Strongyloididae in two different suborders. Such results are similar to those from previous studies [16, 19, 20]. Moreover, the phylogenetic relationship between Rhabditidae and Diplogasteroidea/Diplogasteridae remains under debate, and some authors suggested that Diplogasteroidea/Diplogasteridae belong to Rhabditidae [12, 17, 20]. The present results supported the allocation of Diplogasteroidea/Diplogasteridae within Rhabditidae/Rhabditoidea.

A recent classification placed Ancylostomatidae in Strongyloidea, dissolving the superfamily Ancylostomatoidea [23]. The present results supported this classification, but the monophyly of the family Ancylostomatidae was not supported, similar to the previous studies [52, 60, 61]. The mitogenomic phylogenies also indicated that the Strongyloidea is closer to Trichostrongyloidea than Metastrongyloidea.

## Conclusions

The detailed morphology of *R. kafunata* and *R. bufonis* was further studied using light and scanning electron microscopy. The complete mitochondrial genomes of *R. kafunata* and *R. bufonis* were reported for the first time. The gene arrangement in the mitogenomes of *R. kafunata* and *R. bufonis* represented two new types in Nematoda. The mitogenomic phylogenies indicated that the family Rhabdiasidae is a member of the superfamily Rhabditoidea in Rhabditina, and is closely related to the family Rhabditidae. Molecular phylogenies based on the ITS + 28S sequence data supported the validity of *Kurilonema*, and showed that *Kurilonema* is sister to *Neoentomelas*. The phylogenetic results also indicated that the ancestors of rhabdiasids seem to have infected reptiles first, prior to amphibians.

### Supplementary Information

**Additional file 1: Table S1.** The primers and cycling conditions for amplifying different target regions of *Rhabdias* nematodes by polymerase chain reaction (PCR) in the present study*.*

**Additional file 2: Table S2.** Detailed information on the representatives of Rhabdiasidae with their genetic data included in the phylogenetic analyses.

**Additional file 3: Table S3.** Detailed information on the representatives of Tylenchina and Rhabditina with their mitogenomic data included in the phylogenetic analyses.

**Additional file 4: Table S4.** The partitioning schemes and the optimal models selected for each combination of partition for the BI inference.

**Additional file 5: Table S5.** Annotations and gene organization of *Rhabdias kafunata*. Positive number in the “Gap or overlap” column indicates the length of intergenic sequence, and the negative number indicates the length (absolute number) that adjacent genes overlap (negative sign). The forward strand is marked as “+” and the reverse strand as “−.”

**Additional file 6: Table S6.** Annotations and gene organization of *Rhabdias bufonis.* A positive number in the “Gap or overlap” column indicates the length of intergenic sequence, and the negative number indicates the length (absolute number) that adjacent genes overlap (negative sign). The forward strand is marked as “+” and the reverse strand as “−”.

**Additional file 7: Figure S1.** RSCU of *Rhabdias kafunata* and *R. bufonis*. Codon families (in alphabetical order, from left to right) are provided below the horizontal axis. Values at the top of each bar represent amino acid usage in percentage. RSCU, relative synonymous codon usage.

**Additional file 8: Figure S2.** Inferred secondary structures of 22 tRNAs in the mitogenome of *Rhabdias kafunata*. Lines between bases indicate Watson–Crick bonds, dots indicate GU bonds, and bases in red represent anticodons. tRNA, transfer RNA.

**Additional file 9: Figure S3.** Inferred secondary structures of 22 tRNAs in the mitogenome of *Rhabdias bufonis*. Lines between bases indicate Watson–Crick bonds, dots indicate GU bonds, and bases in red represent anticodons. tRNA, transfer RNA.

## Data Availability

The nuclear and mitochondrial DNA sequences of *Rhabdias kafunata* and *R. bufonis* obtained in the present study were deposited in GenBank database (sequences of *Rhabdias kafunata* under the accession numbers: OR682285–OR682288 (28S), OR682645–OR682648 (ITS), OR685545–OR685548 (*cox1*), OR711464–OR711466 (12S), OR725305 (mitochondrial genome); sequences of *R. bufonis* under the accession numbers: OR690323–OR690327 (28S), OR690329–OR690333 (ITS), OR690336–OR690340 (*cox1*), OR711500–OR711503 (12S), OR725306 (mitochondrial genome). Voucher specimens (20 females of *Rhabdias kafunata* under accession number HBNU–N-2023A1212-ZL, 100 females of *R. bufonis* under accession number HBNU–N-2023A1210-ZL) were deposited in College of Life Sciences, Hebei Normal University, Hebei Province, China.

## Abbreviations

PCG: Protein-coding gene
ML: Maximum likelihood
BI: Bayesian inference
BIC: Bayesian information criterion
28S: Large ribosomal subunit
ITS: Internal transcribed spacer
*cox1*: Cytochrome *c* oxidase subunit 1
12S: 12S small subunit ribosomal RNA gene
SEM: Scanning electron microscopy
RSCU: Relative synonymous codon usage
CAI: Codon Adaptation Index
BS: Bootstrap support
BPP: Bayesian posterior probabilities
