# Supplementary material for: Molecular phylogeny of the family Rhabdiasidae (Nematoda: Rhabditida), with morphology, genetic characterization and mitochondrial genomes of Rhabdias kafunata and R. bufonis
Source: Parasit Vectors. 2024 Mar 1;17:100. doi: 10.1186/s13071-024-06201-z (PMC10908064; doi:10.1186/s13071-024-06201-z)
Supplement: Supplementary file 1 — Additional file 1: Table S1. The primers and cycling conditions for amplifying different target regions of Rhabdias nematodes by polymerase chain reaction (PCR) in the present study. [file 13071_2024_6201_MOESM1_ESM.docx]

**Additional file 1: Table S1.** The primers and cycling conditions for amplifying different target regions of *Rhabdias* nematodes by polymerase chain reaction (PCR) in the present study*.*

| **Primer** | **Sequence 5'-3'** | **Cycling condition** | **Source** |
| --- | --- | --- | --- |
| ITS regions  (ITS-1+5.8S+ITS-2) | #93: 5′ - TTGAACCGGGTAAAAGTCG - 3′  #94: 5′ - TTAGTTTCTTTTCCTCCGCT - 3′ | 94°C for 3min  94°C for 30s  54°C for 30s  72°C for 60s (35 cycles)  72°C for 7min | [1] |
| 28S | #500: 5′ - ACTTTGAAGAGAGAGTTCAAGAG - 3′  #501: 5′ - TCGGAAGGAACCAGCTACTA - 3′ | 94°C for 3min  94°C for 30s  54°C for 30s  72°C for 60s (35 cycles)  72°C for 7min | [1] |
| *cox*1 | LCO1490: 5′ - GGTCAACAAATCATAAAGATATTGG - 3′  HCO2198: 5′ - TAAACTTCAGGGTGACCAAAAAATCA - 3′ | 95°C for 3min  95°C for 30s  50°C for 30s  72°C for 90s (45 cycles)  72°C for 10min | [2] |
| 12S | 12S-F: 5′ - GTTCCAGAATAATCGGCTA - 3′  12S-R: 5′ - ATTGACGGATG(AG)TTTGTACC - 3′ | 94°C for 3 min  94°C for 45 s  48°C for 45 s  72°C for 1 min (35 cycles)  72°C for 5 min | [3] |

**References**

1. Dare OK, Nadler SA & Forbes MR. Nematode lung-worms of two species of anuran amphibians: Evidence for co-adaptation. Int J Parasitol. 2008;38(14):1729–1736.
2. Folmer O, Black M, Hoeh W, Lutz R and Vrijenhoek R. DNA primers for amplification of mitochondrial cytochrome *c* oxidase subunit I from diverse metazoan invertebrates. Mol Mar Biol Biotech. 1994;3(5):294–299.
3. Casiraghi M, Bain O, Guerrero R, et al. Mapping the presence of *Wolbachia pipientis* on the phylogeny of filarial nematodes: evidence for symbiont loss during evolution. Int J Parasitol. 2004;34(2):191–203.
