# Supplementary material for: Molecular phylogeny of the family Rhabdiasidae (Nematoda: Rhabditida), with morphology, genetic characterization and mitochondrial genomes of Rhabdias kafunata and R. bufonis
Source: Parasit Vectors. 2024 Mar 1;17:100. doi: 10.1186/s13071-024-06201-z (PMC10908064; doi:10.1186/s13071-024-06201-z)
Supplement: Supplementary file 2 — Additional file 2: Table S2. Detailed information on the representatives of Rhabdiasidae with their genetic data included in the phylogenetic analyses. [file 13071_2024_6201_MOESM2_ESM.docx]

**Additional file 2: Table S2.** Detailed information on the representatives of Rhabdiasidae with their genetic data included in the phylogenetic analyses.

| **Species** | **Host** | **Locality** | **GenBank ID for ITS region** | **GenBank ID for 28S region** | **References** |
| --- | --- | --- | --- | --- | --- |
| **Ingroup** |  |  |  |  |  |
| **Rhabdiasidae** |  |  |  |  |  |
| ***Rhabdias*** |  |  |  |  |  |
| *R.* *africanus* | *Sclerophrys gutturalis* (Amphibia: Bufonidae) | sub-Saharan Africa | MG428407 | MG428407 | [1] |
| *R. cf. africanus* | *Hylarana galamensis* (Amphibia: Ranidae) | Nigeria | KF999598 | KF999598 | [2] |
| *R. ambystomae* | *Ambystoma maculatum* (Amphibia: Ambystomatidae) | USA | KF999590 | KF999590 | [2] |
| *R. americanus* | *Anaxyrus americanus* (Amphibia: Bufonidae) | USA | KF999589 | KF999589 | [2] |
| *R. bakeri* | *Lithobates sylvatica* (Amphibia: Ranidae) | USA | DQ264770 | DQ264770 | [3] |
| *R. bufonis* | *Rana temporaria* (Amphibia: Ranidae) | Ukraine | KF999593 | KF999593 | [2] |
| *R. cf. bufonis* | *Bombina bombina* (Amphibia: Discoglossidae) | Ukraine | KF999606 | KF999606 | [2] |
| *R.* *bulbicauda* | *Bufo* sp. (Amphibia: Bufonidae) | Nepal | KF999600 | KF999600 | [2] |
| *R. bermani* | *Salamandrella Keyserlingii* (Amphibia: Hynobiidae) | Russia | KF999610 | KF999610 | [2] |
| *R. breviensis* | *Leptodactylus fuscus* (Amphibia: Leptodactylidae) | Brazil | MH516070 | MH516106 | [4] |
| *R.* *delangei* | *Strongylopus grayii* (Amphibia: Pyxicephalidae) | South Africa | MT298095 | MT298095 | [5] |
| *R.* *elegans* | *Bufo* sp. (Amphibia: Bufonidae) | Argentina | KF999604 | KF999604 | [2] |
| *R. engelbrechti* | *Phrynomantis bifasciatus* (Amphibia: Microhylidae) | South Africa | MG428406 | MG428406 | [1] |
| *R. fuelleborni* | *Rhinella diptycha* (Amphibia: Bufonidae) | Brazil | OP651065 | OP651188 | [6] |
| *R. guaianensis* | *Leptodactylus podicipinus* (Amphibia: Leptodactylidae) | Brazil | OP972545 | OP972542 | [7] |
| *R. cf. hylae* | *Litoria pallida* (Amphibia: Hylidae) | Australia | EU836863 | EU836863 | [8] |
| *R. joaquinensis* | *Lithobates blairi* (Amphibia: Ranidae) | USA | KF999594 | KF999594 | [2] |
| *R. cf. joaquinensis* | *Lithobates clamitans* (Amphibia: Ranidae) | USA | KF999608 | KF999608 | [2] |
| *R. kongmonthaensis* | *Polypedates leucomystax* (Amphibia: Rhacophoridae) | Thailand | KF999599 | KF999599 | [2] |
| *R. matogrosensis* | *Leptodactylus macrosternum* (Amphibia: Leptodactylidae) | Brazil | OP972546 | OP972541 | [7] |
| *R.* *nipponica* | *Rana japonica* (Amphibia: Ranidae) | Japan | AB818379 | LC671705 | [9]; [10] |
| *R. nicaraguensis* | *Norops* sp. (Reptilia: Iguanidae) | Costa Rica | KF999605 | KF999605 | [2] |
| *R.* *pseudosphaerocephala* | *Rhinella schneideri* (Amphibia: Bufonidae) | Brazil | MH516078 | MH516078 | [4] |
| *R.* *picardiae* | *Amietia delalandii* (Amphibia: Pyxicephalidae) | South Africa | MG195567 | MG195567 | [11] |
| *R. ranae* | *Rana pipiens* (Amphibia: Ranidae) | USA | DQ264766 | DQ264766 | [3] |
| *R.* *rubrovenosa* | *Bufotes viridis* (Amphibia: Bufonidae) | Ukraine | KF999596 | KF999596 | [2] |
| *R. sphaerocephala* | *Bufo bufo* (Amphibia: Bufonidae) | Ukraine | DQ845739 | DQ845739 | [12] |
| *R. cf. stenocephala* | *Leptodactylus vastus* (Amphibia: Leptodactylidae) | Brazil | MH516077 | MH516113 | [4] |
| *R.* *sylvestris* | *Breviceps sylvestris* (Amphibia: Brevicipitidae) | South Africa | KJ018777 | KJ01877 | [13] |
| *R.* *tarichae* | *Taricha granulosa* (Amphibia: Salamandridae) | California | OL652879 | OL652879 | Unpublish |
| *R. kafunata* | *Bufo gargarizans* (Amphibia: Bufonidae) | China | OR682645 | OR682285 | Present study |
| *R. bufonis* | *Bufo gargarizans* (Amphibia: Bufonidae) | China | OR690331 | OR690325 | Present study |
| ***Entomelas*** |  |  |  |  |  |
| *E. entomelas* | *Anguis fragilis* (Reptilia: Anguidae) | Ukraine | KF999592 | KF999592 | [2] |
| *E.* *kazakhstanica* | *Pseudopus apodus* (Reptilia: Anguidae) | Ukraine | KF999597 | KF999597 | [2] |
| *E.* *ophisauri* | *Pseudopus apodus* (Reptilia: Anguidae) | Ukraine | KF999595 | KF999595 | [2] |
| *E. dujardini* | *Anguis fragilis* (Reptilia: Anguidae) | Ukraine | KF999591 | KF999591 | [2] |
| ***Pneumonema*** |  |  |  |  |  |
| *P. tiliquae* | *Tiliqua scincoides* (Reptilia: Scincidae) | Australia | KF999611 | KF999611 | [2] |
| *Pneumonema* sp. 1 | *Tiliqua scincoides* (Reptilia: Scincidae) | Australia | KF999603 | KF999603 | [2] |
| *Pneumonema* sp. 2 | *Cyclodomorphus Gerrardii* (Reptilia: Scincidae) | Australia | KF999612 | KF999612 | [2] |
| ***Serpentirhabdias*** |  |  |  |  |  |
| *S. fuscovenosa* | *Natrix natrix* (Reptilia: Colubridae) | Ukraine | KF999588 | KF999588 | [2] |
| *S. cf. fuscovenosa* | *Nerodia erythrogaster* (Reptilia: Colubridae) | USA | MH283885 | KF999613 | [14]  [2] |
| *S. elaphe* | *Zamenis longissimus* (Reptilia: Colubridae) | Ukraine | MH283884 | KF999614 | [14]  [2] |
| *S. viperidicus* | *Boothrops moojeni* (Reptilia: Colubridae) | Brazil | MH516095 | KX354358 | [4]  [15] |
| *S. moi* | *Chironius Exoletus* (Reptilia: Colubridae) | Brazil | MH283886 | MH283886 | [14] |
| *S. mussuranae* | *Clelia Clelia* (Reptilia: Colubridae) | Brazil | MK680941 | MK680941 | [16] |
| ***Neoentomelas*** |  |  |  |  |  |
| *N. asatoi* | *Ateuchosaurus pellopleurus* (Reptilia: Scincidae) | Japan | LC631539 | LC631539 | [17] |
| ***Kurilonema*** |  |  |  |  |  |
| *K. markovi* | *Plestiodon* spp. (Reptilia: Scincidae) | Japan | LC631542 | LC631542 | [17] |
| **Outgroup** |  |  |  |  |  |
| *Caenorhabditis elegans* | *Megophrys montana* (Amphibia: Pelobatidae) | San Diego | EF417141 | FJ589007 | [18]  [19] |

**References**

1. Kuzmin Y, Halajian A, Tavakol S, et al. Description and phylogenetic position of a new species of *Rhabdias* Stiles et Hassall, 1905 (Nematoda: Rhabdiasidae) from the banded rubber frog, *Phrynomantis bifasciatus* (Smith) (Amphibia: Microhylidae), in South Africa. Folia Parasit. 2017;64:035.
2. Tkach VV, Kuzmin Y, Snyder SD. Molecular insight into systematics, host associations, life cycles and geographic distribution of the nematode family Rhabdiasidae. Int J Parasitol. 2014;44(5):273–284.
3. Tkach VV, Kuzmin Y, and Pulis EE. A new species of *Rhabdias* from lungs of the wood frog, *Rana sylvatica*, in North America: the last sibling of *Rhabdias ranae*? J Parasitol. 2006;92(3):631–636.
4. Müller MI, Morais DH, Costa-Silva GJ, et al. Diversity in the genus *Rhabdias* (Nematoda, Rhabdiasidae): Evidence for cryptic speciation. Zool Scr. 2018;47:595–607.
5. Kuzmin Y, Svitin R, Harnoster F, et al. Description and molecular characterisation of a new nematode species parasitic in the lungs of *Strongylopus grayii* (Smith) (Anura: Pyxicephalidae) in South Africa. Syst Parasitol. 2020;97(4):369–378.
6. Müller MI, Morais DH, da Costa LFST, et al. Revisiting the taxonomy of *Rhabdias fuelleborni* Travassos, 1928 (Nematoda, Rhabdiasidae) with approaches to delimitation of species and notes on molecular phylogeny. Parasitol Int. 2023;92:e102692.
7. Alcantara EP, Müller MI, Úngari LP, et al. Integrative taxonomy in the genus *Rhabdias* Stiles et Hassall, 1905 from anuran in Brazil, description of two new species and phylogenetic analyses. Parasitol Int. 2023;93:e102714.
8. Dubey S, Shine R. Origin of the parasites of an invading species, the Australian cane toad (*Bufo marinus*): are the lungworms Australian or American? Mol Ecol. 2008;17: 4418–4424.
9. Marcaida Arvin Jet B, et al. Phylogeography of *Rhabdias* spp. (Nematoda: Rhabdiasidae) collected from *Bufo* species in Honshu, Shikoku, and Kyushu, Japan including possible cryptic species. Parasitol Int. 2022;90:e102612.
10. Hasegawa H, Sato A, Kai M, Uchida, A. Helminth parasites of bullfrogs *Lithobates catesbeianus* (Shaw, 1802) in Kanto District, Japan, with special reference to those introduced from North America. Jpn J Vet Parasitol. 2013;12(1):1–10.
11. Svitin R, Kuzmin Y, Preez LD. Molecular and morphological characterisation of *Rhabdias picardiae* Junker, Lhermitte-Vallarino et Bain, 2010 (Nematoda: Rhabdiasidae) from Delaland's River Frog, *Amietia delalandii* (Duméril et Bibron, 1841) (Amphibia: Pyxicephalidae) in South Africa. Acta Parasitologica. 2018;63(1):55–64.
12. Kuzmin Y, Tkach VV, & Brooks DR. Two new species of *Rhabdias* (Nematoda: Rhabdiasidae) from the marine toad, *Bufo marinus* (L.) (Lissamphibia: Anura: Bufonidae) in Central America. J Parasitol. 2007;93:159–165.
13. Tkach VV, Halajian A, Kuzmin Y. Phylogenetic affinities and systematic position of *Entomelas sylvestris* Baker, 1982 (Nematoda: Rhabdiasidae), a parasite of *Breviceps sylvestris* FitzSimons (Amphibia: Brevicipitidae) in South Africa. Syst Parasitol. 2014;87(3):293.
14. Machado SA, Kuzmin Y, Tkach VV, et al. Description, biology and molecular characterisation of *Serpentirhabdias moi* n. sp. (Nematoda: Rhabdiasidae) from *Chironius exoletus* (Serpentes: Colubridae) in Brazil. Parasitol Int. 2018;67(6):829–837.
15. Morais DH, Aguiar A, Müller MI, et al. Morphometric and phylogenetic analyses of *Serpentirhabdias viperidicus* n. sp. (Nematoda: Rhabdiasidae) from the lancehead snake *Bothrops moojeni* Hoge, 1966 (Reptilia: Serpentes: Viperidae) in Brazil. J Helminthol. 2016; 91(3):360–370.
16. Kuzmin Y, Tkach VV, Melo FTV. Description, molecular characterization and life cycle of *Serpentirhabdias mussuranae* n. sp. (Nematoda: Rhabdiasidae) from *Clelia clelia* (Reptilia: Colubroidea) in Brazil. J Helminthol. 2019;94(1):1–12.
17. Sata N, Nakano T. Insights into the phylogenetic position and phylogeography of the monospecific skink-parasite genus *Neoentomelas* (Nematoda: Rhabditida: Rhabdiasidae), with special reference to the effects of the reproductive mode on the genetic diversity. Invertebr Syst. 2022;36(1):36–47.
18. Sonnenberg R, Nolte AW, Tautz D. An evaluation of LSU rDNA D1-D2 sequences for their use in species identification. Front Zool. 2007;4(1):e6.
19. Imai MD, Nadler AS, Brenner D et al. Rhabditid nematode-associated *Ophthalmitis* and *Meningoencephalomyelitis* in captive Asian horned frogs (*Megophrys Montana*). J Vet Diagn Invest. 2009;21(4):568–573.
