# Supplementary material for: Molecular phylogeny of the family Rhabdiasidae (Nematoda: Rhabditida), with morphology, genetic characterization and mitochondrial genomes of Rhabdias kafunata and R. bufonis
Source: Parasit Vectors. 2024 Mar 1;17:100. doi: 10.1186/s13071-024-06201-z (PMC10908064; doi:10.1186/s13071-024-06201-z)
Supplement: Supplementary file 3 — Additional file 3: Table S3. Detailed information on the representatives of Tylenchina and Rhabditina with their mitogenomic data included in the phylogenetic analyses. [file 13071_2024_6201_MOESM3_ESM.docx]

**Additional file 3: Table S3.** Detailed information on the representatives of Tylenchina and Rhabditina with their mitogenomic data included in the phylogenetic analyses.

| **Species** | **Accession** | **Length** | **A+T%** | **References** |
| --- | --- | --- | --- | --- |
| **Ingroup** |  |  |  |  |
| **Rhabditida** |  |  |  |  |
| **Tylenchina** |  |  |  |  |
| **Steinernematoidea** |  |  |  |  |
| **Steinernematidae** |  |  |  |  |
| *Steinernema carpocapsae* | NC_005941 | 13925 | 76.3 | [1] |
| **Aphelenchoidoidea** |  |  |  |  |
| **Aphelenchoididae** |  |  |  |  |
| *Aphelenchoides besseyi* | NC_025291 | 16216 | 80.0 | [2] |
| *Bursaphelenchus xylophilus* | NC_023208 | 14778 | 83.8 | [3] |
| *Bursaphelenchus mucronatus* | NC_021120 | 14583 | 85.3 | [4] |
| **Strongyloidoidea** |  |  |  |  |
| **Strongyloididae** |  |  |  |  |
| *Strongyloides vituli* | NC_066507 | 14624 | 71.5 | [5] |
| *Strongyloides cebus* | NC_066659 | 16948 | 77.8 | [5] |
| **Cephaloboidea** |  |  |  |  |
| **Cephalobidae** |  |  |  |  |
| *Acrobeles complexus* | KM192361 | 14780 | 63.8 | [6] |
| **Tylenchoidea** |  |  |  |  |
| **Meloidogynidae** |  |  |  |  |
| *Meloidogyne graminicola* | NC_056772 | 19589 | 83.5 | [7] |
| *Meloidogyne javanica* | NC_026556 | 182391 | 83.4 | [8] |
| **Heteroderidae** |  |  |  |  |
| *Heterodera glycines* | HM640930 | 14915 | 82.7 | [9] |
| *Globodera ellingtonae* | KU726972 | 14365 | 67.5 | [10] |
| **Pratylenchidae** |  |  |  |  |
| *Pratylenchus vulnus* | GQ332425 | 21656 | 73.8 | [3] |
| **Rhabditina** |  |  |  |  |
| **Rhabditoidea** |  |  |  |  |
| **Mesorhabditidae** |  |  |  |  |
| *Cruznema tripartitum* | NC_072121 | 14067 | 78.7 | [11] |
| **Rhabditidae** |  |  |  |  |
| *Caenorhabditis tropicalis* | NC_025756 | 13874 | 75.7 | [12] |
| *Caenorhabditis elegans* | NC_001328 | 13794 | 76.2 | [13] |
| **Rhabdiasidae** |  |  |  |  |
| *Rhabdias kafunata* | OR725305 | 15437 | 75.8 | Present study |
| *Rhabdias bufonis* | OR725306 | 15128 | 76.7 | Present study |
| **Diplogastridae** |  |  |  |  |
| *Allodiplogaster sudhausi* | NC_029233 | 16005 | 77.5 | [14] |
| *Pristionchus pacificus* | NC_015245 | 15954 | 76.2 | [15] |
| **Metastrongyloidea** |  |  |  |  |
| **Metastrongylidae** |  |  |  |  |
| *Metastrongylus salmi* | NC_013815 | 13778 | 74.0 | [16] |
| *Metastrongylus pudendotectus* | NC_013813 | 13793 | 77.5 | [16] |
| *Dictyocaulus viviparus* | NC_019810 | 13310 | 76.4 | [17] |
| *Dictyocaulus eckerti* | NC_019809 | 13300 | 76.3 | [17] |
| *Aelurostrongylus abstrusus* | NC_019571 | 13913 | 71.9 | [18] |
| *Protostrongylus rufescens* | NC_023262 | 13619 | 74.6 | [19] |
| *Varestrongylus eleguneniensis* | NC_068834 | 13625 | 75.9 | [20] |
| **Filaroididae** |  |  |  |  |
| *Parafilaroides normani* | NC_024656 | 13414 | 72.2 | [21] |
| **Trichostrongyloidea** |  |  |  |  |
| **Trichostrongylidae** |  |  |  |  |
| *Trichostrongylus axei* | NC_013824 | 13653 | 76.4 | [16] |
| *Trichostrongylus vitrinus* | NC_013807 | 13800 | 79.0 | [16] |
| *Cooperia oncophora* | NC_004806 | 13636 | 77.4 | [22] |
| *Haemonchus contortus* | NC_010383 | 14055 | 78.1 | [23] |
| *Marshallagia marshalli* | NC_036409 | 13891 | 76.2 | [24] |
| *Mecistocirrus digitatus* | NC_013848 | 15221 | 79.7 | [16] |
| *Teladorsagia circumcincta* | NC_013827 | 14066 | 77.1 | [16] |
| **Molineidae** |  |  |  |  |
| *Nematodirus oiratianus* | NC_024639 | 13765 | 76.1 | [25] |
| *Nematodirus spathiger* | NC_024638 | 13519 | 75.0 | [25] |
| **Heligmonellidae** |  |  |  |  |
| *Nippostrongylus brasiliensis* | NC_033886 | 13355 | 76.4 | [26] |
| **Strongyloidea** |  |  |  |  |
| **Ancylostomatidae** |  |  |  |  |
| *Necator americanus* | NC_003416 | 13605 | 76.6 | [27] |
| *Ancylostoma tubaeforme* | NC_034289 | 13730 | 77.8 | [28] |
| *Ancylostoma caninum* | NC_012309 | 13717 | 77.5 | [29] |
| *Uncinaria sanguinis* | NC_025267 | 13753 | 78.9 | [30] |
| *Bunostomum phlebotomum* | NC_012308 | 13790 | 77.1 | [29] |
| **Syngamidae** |  |  |  |  |
| *Syngamus trachea* | NC_013821 | 14647 | 73.8 | [16] |
| **Strongylidae** |  |  |  |  |
| *Strongylus vulgaris* | NC_013818 | 14301 | 76.6 | [16] |
| *Coronocyclus labratus* | NC_061656 | 13856 | 76.9 | [31] |
| *Chabertia erschowi* | NC_023782 | 13705 | 74.4 | [32] |
| *Chabertia ovina* | NC_013831 | 13682 | 76.3 | [16] |
| *Hypodontus macropi* | NC_023098 | 13634 | 73.3 | [33] |
| *Macropicola ocydromi* | NC_023099 | 13659 | 75.8 | [33] |
| *Macropostrongyloides dissimilis* | MW309879 | 13691 | 76.3 | [34] |
| *Macropostrongyloides woodi* | MW309878 | 13847 | 77.2 | [34] |
| *Paramacropostrongylus typicus* | OK111107 | 13723 | 76.8 | [35] |
| *Paramacropostrongylus iugalis* | OK111106 | 13713 | 76.8 | [35] |
| *Phascolostrongylus turleyi* | OK111104 | 13905 | 75.6 | [35] |
| *Torquenema toraliforme* | OK111108 | 13764 | 77.0 | [35] |
| *Oesophagostomoides stirtoni* | OK111103 | 13843 | 76.3 | [35] |
| *Oesophagostomoides longispicularis* | OK111102 | 13719 | 75.3 | [35] |
| *Stephanurus dentatus* | MW970029 | 13735 | 71.6 | [36] |
| **Outgroup** |  |  |  |  |
| *Toxocara cati* | NC_010773 | 14029 | 69.9 | [37] |

**References**

1. Montiel R, Lucena MA, Medeiros J, et al. The complete mitochondrial genome of the entomopathogenic nematode *Steinernema carpocapsae*: insights into nematode mitochondrial DNA evolution and phylogeny. J Mol Evol. 2006;62(2):e211.
2. Sun L, Chi W, Zhuo K, et al. The complete mitochondrial genome of *Aphelenchoides besseyi* (Nematoda: Aphelenchoididae), the first sequenced representative of the subfamily Aphelenchoidinae. Nematology. 2014;16(10):1167–1180.
3. Sultana T, Kim J, Lee SH, et al. Comparative analysis of complete mitochondrial genome sequences confirms independent origins of plant-parasitic nematodes. BMC Evol Biol. 2013;13(1):e12.
4. Sultana T, Han H, Park JK. Comparison of complete mitochondrial genomes of pine wilt nematode *Bursaphelenchus xylophilus* and *Bursaphelenchus mucronatus* (Nematoda: Aphelenchoidea) and development of a molecular tool for species identification. Gene. 2013;520(1):39–46.
5. Ko PP, Haraguchi M, Hara T, et al. Population genetics study of *Strongyloides fuelleborni* and phylogenetic considerations on primate-infecting species of *Strongyloides* based on their mitochondrial genome sequences. Parasitol Int. 2023;92:e102663.
6. Jiyeon, Kim, Sang-Hwa, et al. Mitochondrial genomes advance phylogenetic hypotheses for Tylenchina (Nematoda: Chromadorea). Zool Scr. 2015;44(4):446–462.
7. Sun LH, Zhuo K, Lin BR, et al. The complete mitochondrial genome of *Meloidogyne graminicola* (Tylenchina): A unique gene arrangement and its phylogenetic implications. Plos One. 2014;9(6):e98558.
8. Humphreys-Pereira DA, Elling AA. Mitochondrial genome plasticity among species of the nematode genus *Meloidogyne* (Nematoda: Tylenchina). Gene. 2015;560(2):173–183.
9. Gibson T, Farrugia D, Barrett J, et al. The mitochondrial genome of the soybean cyst nematode, *Heterodera glycines*. Genome. 2011;54(7):565–574.
10. Phillips WS, Brown AMV, Howe DK, et al. The mitochondrial genome of *Globodera ellingtonae* is composed of two circles with segregated gene content and differential copy numbers. BMC Genom. 2016;17(1):e706.
11. Du HR, Guo F, Gao YX et al. Complete mitogenome of *Cruznema tripartitum* confirms highly conserved gene arrangement within family Rhabditidae. J Nematol. 2022;54(1):20220029.
12. Yang SS, Li S, Wang GX. The complete mitochondrial genome of *Caenorhabditis tropicalis* n. sp. (Rhabditida: Rhabditidae). Mitochondrial DNA A. 2016;27(3):1–2.
13. Wolstenholme DR, Ronald O, Macfarlane JL. Nucleotide correlations that suggest tertiary interactions in the TV-replacement loop-containing mitochondrial tRNAs of the nematodes, *Caenorhabditis elegans* and *Ascaris suum*. Nucleic Acids Res. 1994;22(20):4300–4306.
14. Kim T, Kim J, Nadler SA, Park JK. The complete mitochondrial genome of *Koerneria sudhausi* (Diplogasteromorpha: Nematoda) supports monophyly of Diplogasteromorpha within Rhabditomorpha. Curr Genet. 2016;62:391–403.
15. Sommer RJ. Mutation rates and intraspecific divergence of the mitochondrial genome of *Pristionchus pacificus*. Mol Biol & Evol. 2011;28(8):2317–26.
16. Jex AR, Hall RS, Littlewood DTJ, et al. An integrated pipeline for next-generation sequencing and annotation of mitochondrial genomes. Nucleic Acids Res. 2009;38(2):522–533.
17. Gasser RB, Jabbar A, Mohandas N, et al. Assessment of the genetic relationship between *Dictyocaulus* species from *Bos taurus* and *Cervus elaphus* using complete mitochondrial genomic datasets. Parasite Vector. 2012;5(1):e241.
18. Jabbar A, Jex AR, Mohandas N, et al. The mitochondrial genome of *Aelurostrongylus abstrusus*-diagnostic, epidemiological and systematic implications. Gene. 2013;516(2):294–300.
19. Jabbar A, Mohandas N, Jex AR, et al. The mitochondrial genome of *Protostrongylus rufescens* – implications for population and systematic studies. Parasite Vector. 2013;6(1):e203.
20. Xie Y, Chen YJ, Wang LD, et al. Comprehensive molecular characterization of the mitochondrial genome of the takin lungworm *Varestrongylus eleguneniensis* (Strongylida: Protostrongylidae). Int J Mol Sci. 2022;23(1):e13597.
21. Jabbar A, Mohandas N, Gasser RB. Characterisation of the mitochondrial genome of *Parafilaroides normani* (lungworm) of *Arctocephalus pusillus doriferus* (Australian fur seal). Parasitol Res. 2014;113(8):3049–3055.
22. Van DVM, De Vries E. A single nucleotide polymorphism map of the mitochondrial genome of the parasitic nematode *Cooperia oncophora*. Parasitology. 2004;128(4):421–431.
23. Jex AR, Hu M, Littlewood DTJ, et al. Using 454 technology for long-PCR based sequencing of the complete mitochondrial genome from single *Haemonchus contortus* (Nematoda). BMC Genom. 2008;9(1):1–14.
24. Sun MM, Han L, Zhang FK, et al. Characterization of the complete mitochondrial genome of *Marshallagia marshalli* and phylogenetic implications for the superfamily Trichostrongyloidea. Parasitol Res. 2018;117(1):307–313.
25. Zhao GH, Jia YQ, Cheng WY, et al. Characterization of the complete mitochondrial genomes of *Nematodirus oiratianus* and *Nematodirus spathiger* of small ruminants. Parasit Vectors. 2014;7(1):319.
26. Chandler J, Camberis M, Bouchery T, et al. Annotated mitochondrial genome with Nanopore R9 signal for *Nippostrongylus brasiliensis*. F1000research. 2017;6:56.
27. Hu M, Chilton NB, Gasser RB. The mitochondrial genomes of the human hookworms *Ancylostoma duodenale* and *Necator americanus* (Nematoda: Secernentea). Int J Parasitol. 2002;32(2):145–158.
28. Shi XL, Fu YQ, Abdullahi AY, et al. The mitochondrial genome of *Ancylostoma tubaeforme* from cats in China. J Helminthol. 2018;92(1):22–33.
29. Jex AR, Waeschenbach A, Hu M, et al. The mitochondrial genomes of *Ancylostoma caninum* and *Bunostomum phlebotomum* – two hookworms of animal health and zoonotic importance. BMC Genom. 2009;10(1):e79.
30. Haynes BT, Marcus AD, Higgins DP, et al. Unexpected absence of genetic separation of a highly diverse population of hookworms from geographically isolated hosts. Infect Genet Evol. 2014;28:192–200.
31. Yang S, Li P, Sun C, et al. The complete mitochondrial genome of *Coronocyclus labratus* (Rhabditida: Cyathostominae). Mitochondrial DNA B. 2020;5(1):1044–1045.
32. Liu GH, Zhao L, Song HQ, et al. *Chabertia erschowi* (Nematoda) is a distinct species based on nuclear ribosomal DNA sequences and mitochondrial DNA sequences. Parasite Vector. 2014;7(1):e44.
33. Jabbar A, Beveridge I, Mohandas N, et al. Analyses of mitochondrial amino acid sequence datasets support the proposal that specimens of *Hypodontus macropi* from three species of macropodid hosts represent distinct species. BMC Evol Biol. 2013;13(1):259.
34. Sukee T, Koehler AV, Hall R, et al. Phylogenetic analysis of mitogenomic data sets resolves the relationship of seven *Macropostrongyloides* species from Australian macropodid and vombatid marsupials. Pathogens. 2020;9(12):1–11.
35. Sukee T, Beveridge I, Koehler AV, et al. Phylogenetic relationships of the nematode subfamily Phascolostrongylinae from macropodid and vombatid marsupials inferred using mitochondrial protein sequence data. Parasite Vector. 2021;14(1):e523.
36. Deng YP, Zhang XL, Li LY, et al. Characterization of the complete mitochondrial genome of the swine kidney worm *Stephanurus dentatus* (Nematoda: Syngamidae) and phylogenetic implications. Vet Parasitol. 2021; 295:e109475.
37. Li MW, Lin RQ, Song HQ, et al. The complete mitochondrial genomes for three *Toxocara* species of human and animal health significance. BMC Genom. 2008;9(1):e224.
