# Supplementary material for: Molecular phylogeny of the family Rhabdiasidae (Nematoda: Rhabditida), with morphology, genetic characterization and mitochondrial genomes of Rhabdias kafunata and R. bufonis
Source: Parasit Vectors. 2024 Mar 1;17:100. doi: 10.1186/s13071-024-06201-z (PMC10908064; doi:10.1186/s13071-024-06201-z)
Supplement: Supplementary file 4 — Additional file 4: Table S4. The partitioning schemes and the optimal models selected for each combination of partition for the BI inference. [file 13071_2024_6201_MOESM4_ESM.doc]

| Subset | Best model | Number of site | Partitioning scheme |
| --- | --- | --- | --- |
| 1 | MTREV+G | 245 | *nad*2 |
| 2 | CPREV+G | 218 | *nad*3; *nad*6 |
| 3 | MTREV+G | 71 | *nad*4L |
| 4 | MTREV+I+G | 1244 | *nad*4; *nad*5; *cyt*b |
| 5 | BLOSUM62+G | 189 | *atp*6 |
| 6 | MTREV+I+G | 522 | *cox*1 |
| 7 | CPREV+I+G | 505 | *cox*2; *nad*1 |
| 8 | MTREV+G | 253 | *cox*3 |

**Additional file 4: Table S4.** The partitioning schemes and the optimal models selected for each combination of partition for the BI inference.
