# Supplementary material for: Molecular phylogeny of the family Rhabdiasidae (Nematoda: Rhabditida), with morphology, genetic characterization and mitochondrial genomes of Rhabdias kafunata and R. bufonis
Source: Parasit Vectors. 2024 Mar 1;17:100. doi: 10.1186/s13071-024-06201-z (PMC10908064; doi:10.1186/s13071-024-06201-z)
Supplement: Supplementary file 6 — Additional file 6: Table S6. Annotations and gene organization of Rhabdias bufonis. A positive number in the “Gap or overlap” column indicates the length of intergenic sequence, and the negative number indicates the length (absolute number) that adjacent genes overlap (negative sign). The forward strand is marked as “+” and the reverse strand as “−”. [file 13071_2024_6201_MOESM6_ESM.docx]

**Additional file 6: Table S6.** Annotations and gene organization of *Rhabdias bufonis.* Positive number in the “Gap or overlap” column indicates the length of intergenic sequence, and the negative number indicates the length (absolute number) that adjacent genes overlap (negative sign). The forward strand is marked as “+” and the reverse strand as “-”.

| Gene | Type | Start | End | Length | Start Codon | Stop Codon | Anticodon | Strand | Gap or overlap |
| --- | --- | --- | --- | --- | --- | --- | --- | --- | --- |
| *cox*1 | CDS | 1 | 1512 | 1512 | ATT | TAA |  | + | 56 |
| *nad*3 | CDS | 1569 | 1919 | 351 | ATA | TAA |  | + | 12 |
| tRNA-Pro (P) | tRNA | 1932 | 1987 | 56 |  |  | UGG | + | 58 |
| *cox*2 | CDS | 2046 | 2738 | 693 | ATA | TAG |  | + | 0 |
| tRNA-His (H) | tRNA | 2739 | 2792 | 54 |  |  | GUG | + | 0 |
| *rrn*L | rRNA | 2793 | 3776 | 984 |  |  |  | + | 0 |
| *nad*5 | CDS | 3777 | 5366 | 1590 | ATT | TAA |  | + | 1 |
| tRNA-Ala (A) | tRNA | 5368 | 5423 | 56 |  |  | UGC | + | 0 |
| NCR1 | Non-coding region | 5424 | 6019 | 596 |  |  |  | + | 0 |
| tRNA-Cys (C) | tRNA | 6020 | 6075 | 56 |  |  | GCA | + | 0 |
| NCR2 | Non-coding region | 6076 | 6546 | 471 |  |  |  | + | 0 |
| tRNA-Met (M) | tRNA | 6547 | 6607 | 61 |  |  | CAU | + | 21 |
| tRNA-Val (V) | tRNA | 6629 | 6683 | 55 |  |  | UAC | + | 6 |
| tRNA-Asp (D) | tRNA | 6690 | 6745 | 56 |  |  | GUC | + | 7 |
| tRNA-Gly (G) | tRNA | 6753 | 6809 | 57 |  |  | UCC | + | 49 |
| *nad*6 | CDS | 6859 | 7299 | 441 | ATA | TAA |  | + | 0 |
| NCR3 | Non-coding region | 7300 | 7775 | 476 |  |  |  | + | 0 |
| *nad*4L | CDS | 7776 | 8009 | 234 | ATT | TAA |  | + | 0 |
| tRNA-Trp (W) | tRNA | 8010 | 8064 | 55 |  |  | UCA | + | 6 |
| tRNA-Glu (E) | tRNA | 8071 | 8127 | 57 |  |  | UUC | + | 0 |
| *rrn*S | rRNA | 8128 | 8829 | 702 |  |  |  | + | 0 |
| tRNA-Ser2 (S2) | tRNA | 8830 | 8884 | 55 |  |  | UGA | + | 4 |
| tRNA-Asn (N) | tRNA | 8889 | 8944 | 56 |  |  | GUU | + | 13 |
| tRNA-Tyr (Y) | tRNA | 8958 | 9014 | 57 |  |  | GUA | + | 0 |
| *nad*1 | CDS | 9015 | 9887 | 873 | TTG | TAA |  | + | 1 |
| *atp*6 | CDS | 9889 | 10488 | 600 | ATT | TAA |  | + | 6 |
| tRNA-Lys (K) | tRNA | 10495 | 10556 | 62 |  |  | UUU | + | 4 |
| tRNA-Leu2 (L2) | tRNA | 10561 | 10616 | 56 |  |  | UAA | + | 0 |
| tRNA-Ser1 (S1) | tRNA | 10617 | 10670 | 54 |  |  | UCU | + | 0 |
| *nad*2 | CDS | 10671 | 11516 | 846 | TTG | TAA |  | + | 1 |
| tRNA-Ile (I) | tRNA | 11518 | 11578 | 61 |  |  | GAU | + | 0 |
| tRNA-Arg (R) | tRNA | 11579 | 11632 | 54 |  |  | ACG | + | 4 |
| tRNA-Gln (Q) | tRNA | 11637 | 11691 | 55 |  |  | UUG | + | 3 |
| tRNA-Phe (F) | tRNA | 11695 | 11750 | 56 |  |  | GAA | + | 0 |
| *cyt*b | CDS | 11751 | 12858 | 1108 | TTG | T |  | + | 0 |
| tRNA-Leu1 (L1) | tRNA | 12859 | 12914 | 56 |  |  | UAG | + | 0 |
| *cox*3 | CDS | 12915 | 13682 | 768 | TTG | TAA |  | + | 4 |
| tRNA-Thr (T) | tRNA | 13687 | 13742 | 56 |  |  | UGU | + | 0 |
| *nad*4 | CDS | 13743 | 14972 | 1230 | TTG | TAA |  | + | 0 |
| NCR4 | Non-coding region | 14973 | 15128 | 156 |  |  |  | + |  |
