# Supplementary figures and images for: Molecular phylogeny of the family Rhabdiasidae (Nematoda: Rhabditida), with morphology, genetic characterization and mitochondrial genomes of Rhabdias kafunata and R. bufonis
Source: Parasit Vectors. 2024 Mar 1;17:100. doi: 10.1186/s13071-024-06201-z (PMC10908064; doi:10.1186/s13071-024-06201-z)

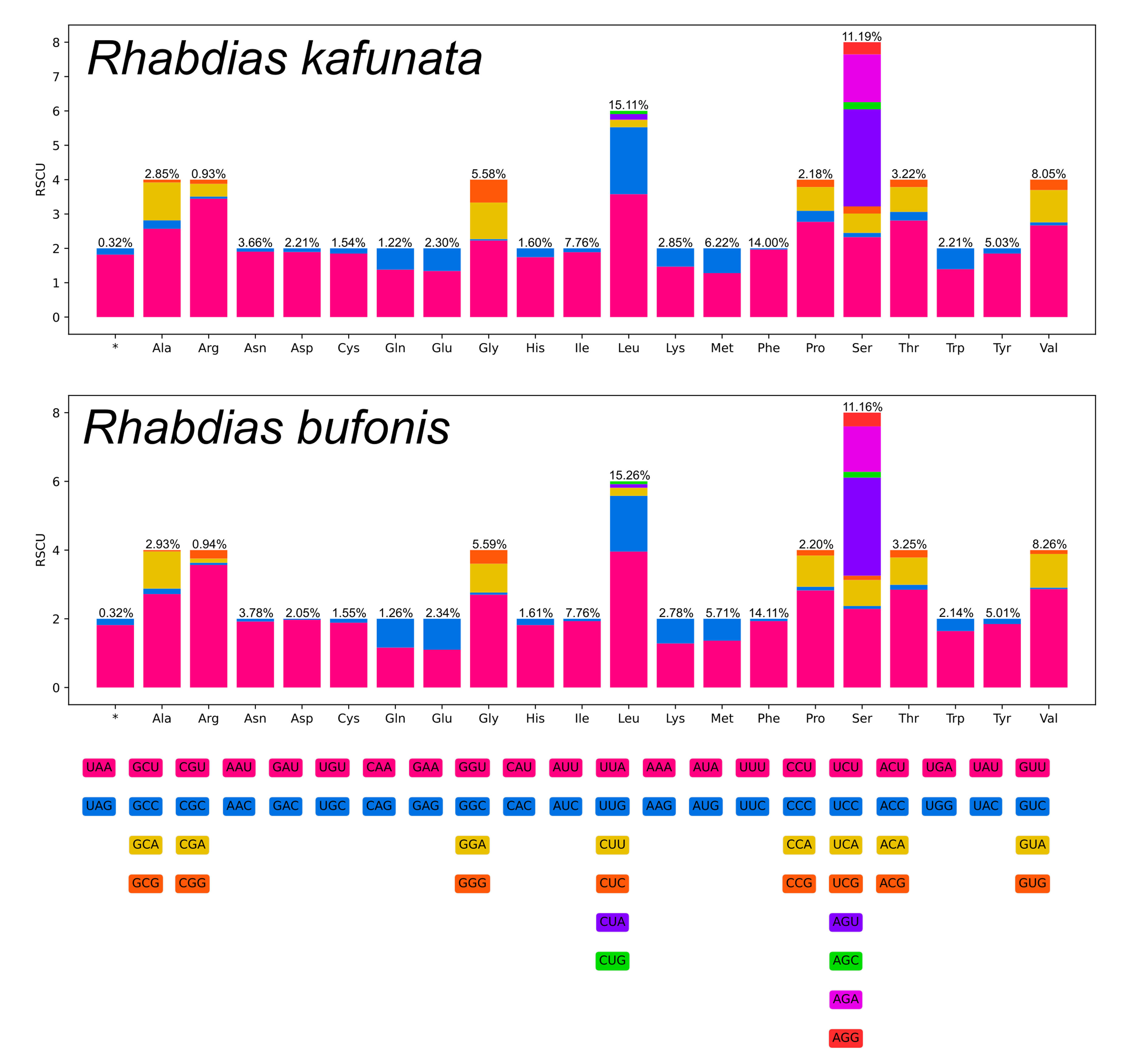

Supplement: Supplementary file 7 — Additional file 7: Figure S1. RSCU of Rhabdias kafunata and R. bufonis. Codon families (in alphabetical order, from left to right) are provided below the horizontal axis. Values at the top of each bar represent amino acid usage in percentage. RSCU, relative synonymous codon usage. [file 13071_2024_6201_MOESM7_ESM.tif]

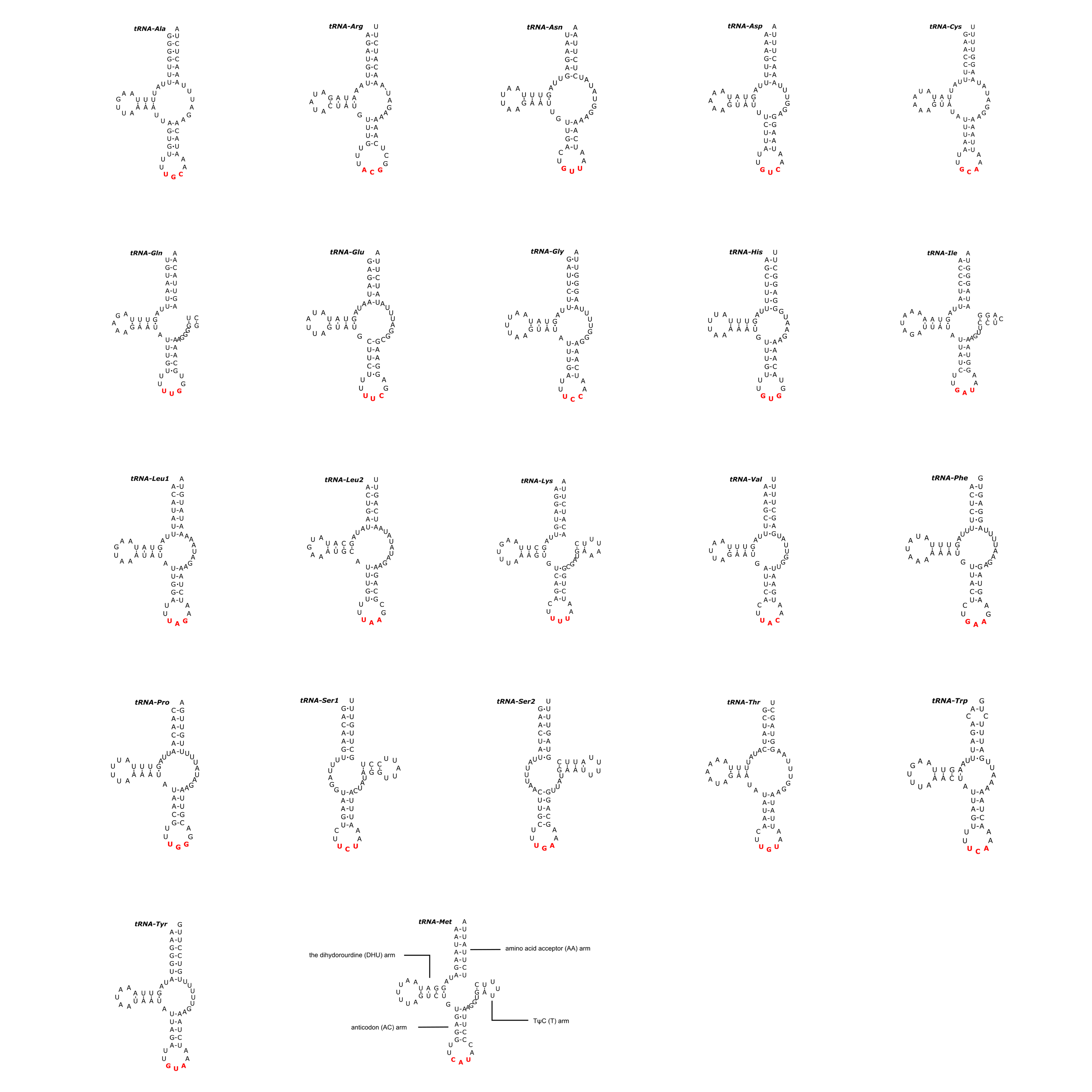

Supplement: Supplementary file 8 — Additional file 8: Figure S2. Inferred secondary structures of 22 tRNAs in the mitogenome of Rhabdias kafunata. Lines between bases indicate Watson–Crick bonds, dots indicate GU bonds, and bases in red represent anticodons. tRNA, transfer RNA. [file 13071_2024_6201_MOESM8_ESM.tif]

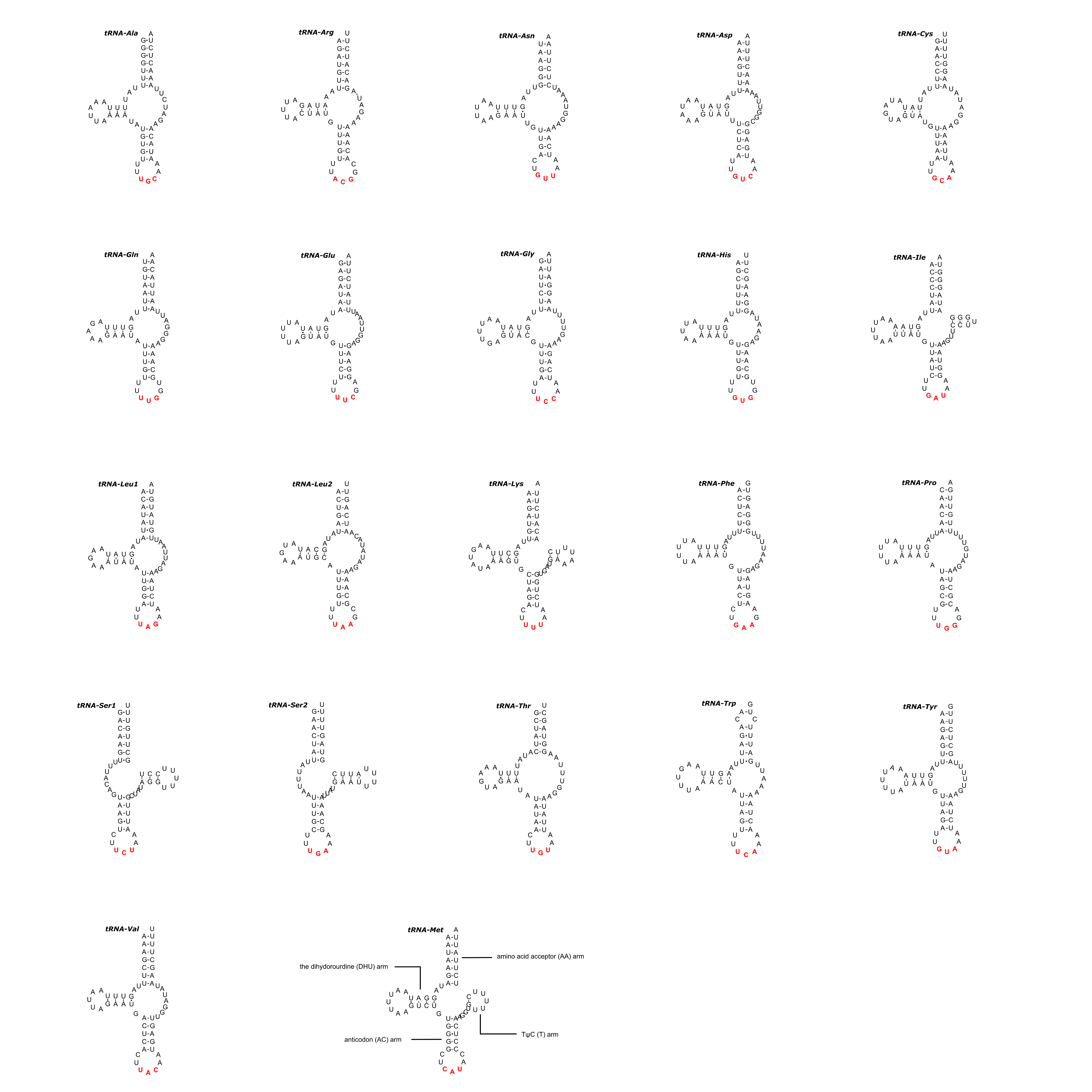

Supplement: Supplementary file 9 — Additional file 9: Figure S3. Inferred secondary structures of 22 tRNAs in the mitogenome of Rhabdias bufonis. Lines between bases indicate Watson–Crick bonds, dots indicate GU bonds, and bases in red represent anticodons. tRNA, transfer RNA. [file 13071_2024_6201_MOESM9_ESM.tif]
